# Collinear Jahn–Teller Ordering Induces Monoclinic Distortion in “Defect-Free” LiNiO_2_

**DOI:** 10.1021/jacs.5c07435

**Published:** 2025-07-31

**Authors:** George S. Phillips, James M. A. Steele, Farheen N. Sayed, Leonhard Karger, Liam A. V. Nagle-Cocco, Annalena R. Genreith-Schriever, Gabriel E. Pérez, David A. Keen, Jürgen Janek, Torsten Brezesinski, Joshua D. Bocarsly, Siân E. Dutton, Clare P. Grey

**Affiliations:** † Yusuf Hamied Department of Chemistry, 2152 University of Cambridge, Lensfield Road, Cambridge CB2 1EW, U.K.; ‡ The Faraday Institution, Didcot OX11 0RA, U.K.; § Cavendish Laboratory, 2152 University of Cambridge, JJ Thomson Avenue, Cambridge CB3 0HE, U.K.; ∥ Battery and Electrochemistry Laboratory (BELLA), Institute of Nanotechnology, Karlsruhe Institute of Technology (KIT), Kaiserstr. 12, 76131 Karlsruhe, Germany; ⊥ ISIS Neutron and Muon Source, Rutherford Appleton Laboratory, Harwell Science and Innovation Campus, Didcot OX11 0QX, U.K.; # Institute of Physical Chemistry & Center for Materials Research (ZfM/LaMa), Justus-Liebig-University Giessen, Heinrich-Buff-Ring 17, 35392 Giessen, Germany; ∇ Department of Chemistry and Texas Center for Superconductivity, 14743 University of Houston, Houston, Texas 77004, United States

## Abstract

Lithium nickel oxide, LiNiO_2_ (LNO), and its doped derivatives are promising battery cathode materials with high gravimetric capacity and operating voltages. They are also of interest to the field of quantum magnetism due to the presumed *S* = 1/2 triangular lattice and associated geometric frustration. However, the tendency for Li/Ni substitutional defects and off-stoichiometry makes fundamental studies challenging. In particular, there is still a discrepancy between the rhombohedral (*R*3̅*m*) bulk structure and the Jahn–Teller (JT) distortions of the NiO_6_ octahedra inferred on the basis of local structural probes. Karger *et al*. (*Chem. Mater.* **2023**, *35*, 648–657) recently used Na/Li ion exchange to synthesize “defect-free” LNO by exploiting the absence of antisite disorder in NaNiO_2_ (NNO). Here we characterize the short- and long-range structure of this ion-exchanged material and observe splittings of key Bragg reflections at 100 K in X-ray and neutron diffraction (XRD and NPD), indicative of a monoclinic distortion induced by a cooperative collinear JT distortion, similar to that seen in NNO. Variable temperature XRD reveals a second-order phase transition from the monoclinic (*C*2/*m*) low-temperature structure to a rhombohedral (*R*3̅*m*) structure above ∼400 K. We propose that this collinear JT ordering is also present in solid-state synthesized LNO with the domain size and extent of monoclinic distortion controlled by defect concentration. This new structural description of LNO will help advance our understanding of its electronic and magnetic properties and the series of phase transformations that this material undergoes upon electrochemical cycling in Li-ion batteries.

## Graphical abstract

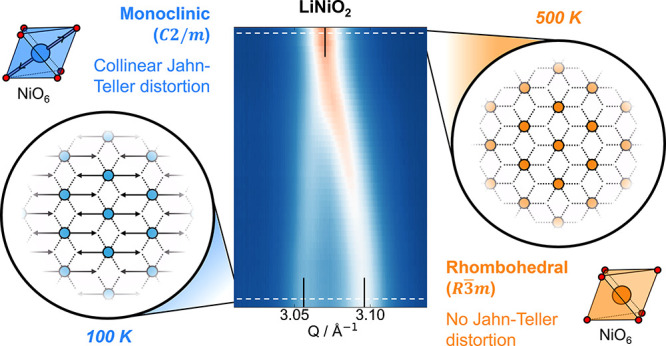

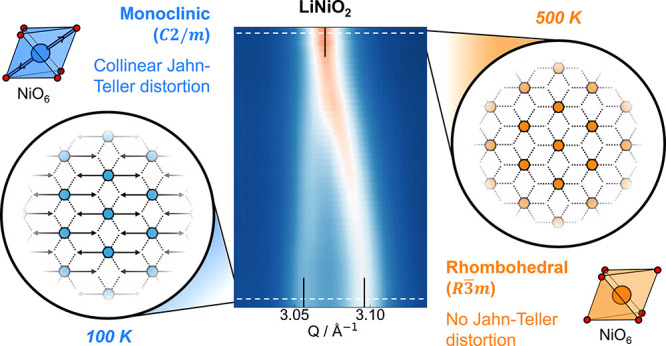

## Introduction

Lithium nickel oxide (LiNiO_2_ or LNO) has been widely studied as a potential battery cathode material as it represents the “cobalt-free” end member of a series of commercially important layered nickel manganese cobalt (NMC) oxides with composition Li­(Ni_1‑*x*‑*y*_Mn_*x*_Co_*y*_)­O_2_.^1–3^ While NMC811 (the phase with composition *x*,*y* = 0.1) is currently used in many electric vehicles, there is a push to remove Co entirely due to its higher cost and ethical concerns with its mining, motivating studies of materials with increasingly high Ni content. However, reducing the extent of transition-metal doping toward LNO limits the solid-solution behavior on de/lithiation and introduces phase transitions with large volume changes, likely driven by a combination of cation- and charge-ordering, and different degrees of Jahn–Teller (JT) ordering of the Ni^3+^ ion with its d^7^ electron configuration.^4–6^

LNO adopts the α-NaFeO_2_ structure, with the Li^+^ and Ni^3+^ cations occupying alternating (111) planes of a cubic rock-salt structure. The oxygen anions form an interpenetrated close-packed face-centered cubic (FCC) lattice, resulting in edge-sharing octahedral cation sites (Figure 1a).^7^ The average structure of LNO is reported to be rhombohedral, adopting the *R*3̅*m* space group based on diffraction measurements. However, studies of the local structure show distorted NiO_6_ octahedra and a splitting of the Ni–O bond lengths, with several structural models presented in the literature, but no consensus reached.^8,9^ The Ni–O bond length splitting has been ascribed in some studies to JT distortions arising from the d^7^ Ni^3+^ ion, with collinear and zigzag orderings (Figure 1b) calculated as almost degenerate ground-states from density functional theory.^10–14^ Alternatively, a charge-disproportionated model has been suggested to account for the Ni–O bond lengths splitting via expanded Ni^II^O_6_ and contracted Ni^IV^O_6_ octahedra.^15–17^ By contrast NaNiO_2_ (NNO) has a well understood layered structure with cooperative collinear JT distortions of the Ni^3+^ ions resulting in a monoclinic structure belonging to the *C*2/*m* space group at room temperature.^18^ On heating, NNO undergoes a first-order displacive phase transition to adopt a *R*3̅*m* high-temperature structure, isostructural with room temperature LNO, with a mixed phase regime from 460 to 505 K.^19,20^ The equivalent monoclinic and rhombohedral cells are highlighted in Figure 1a, where the latter cell is depicted as a trigonal cell within a hexagonal setting.

**Figure 1:**
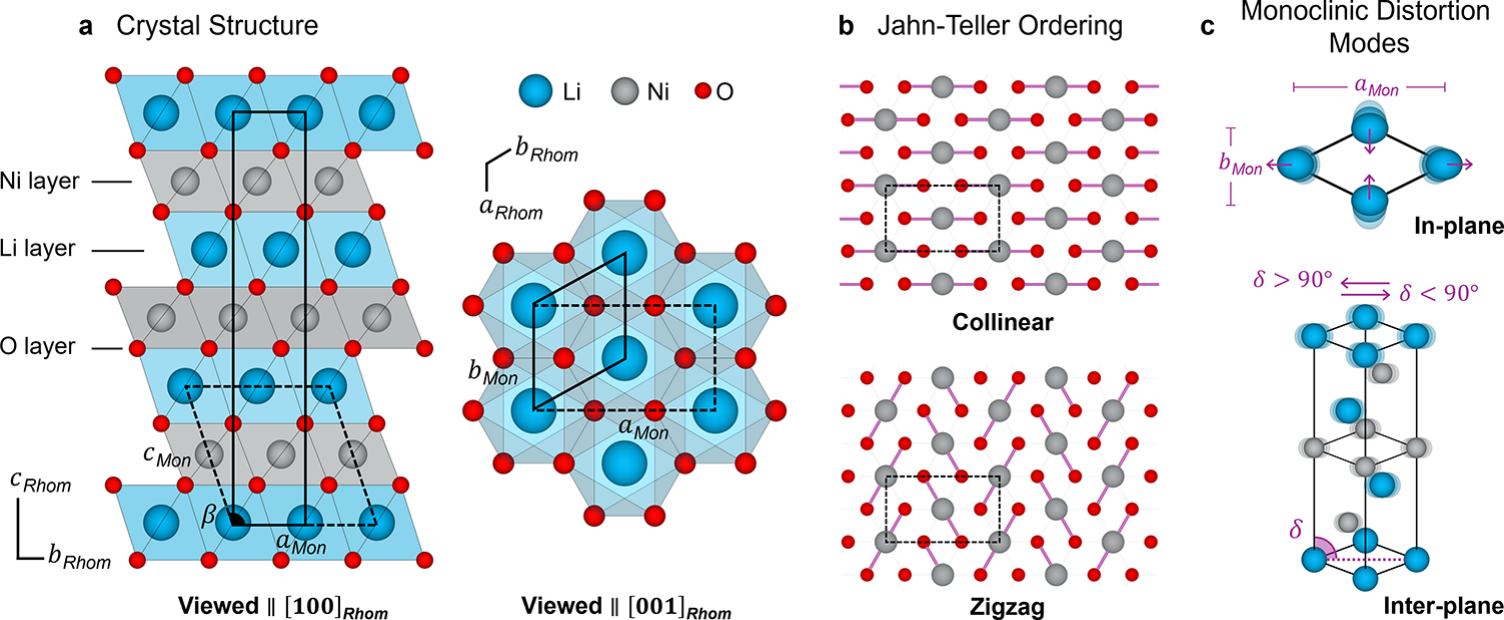
Crystal structure of LiNiO_2_, cooperative Jahn–Teller ordering schemes, and monoclinic distortion modes. (a) Structure of LNO viewed from parallel to [100]_Rhom_ and [001]_Rhom_. Lithium ions are shown in blue, nickel in gray, and oxygen in red, with the LiO_6_ and NiO_6_ octahedra colored accordingly. The rhombohedral (*R*3̅*m*) and monoclinic unit cells are shown with a solid black line and dashed lines, respectively. (b) Collinear (top) and zigzag (bottom) Jahn–Teller orderings, viewed perpendicular to the *ab*-plane, with the unit cell indicated with dashed black lines. Only elongated O–Ni–O bonds are shown (as purple lines). (c) Two orthogonal monoclinic distortion modes: the in-plane variation of the *a*_Mon_/*b*_Mon_ ratio (top) and interplane shear quantified by the delta angle (bottom, as introduced by Chung *et al*.^8^).

The extent of distortion from the rhombohedral symmetry structure can be parametrized by two orthogonal monoclinic distortion modes: the in-plane variation of the *a*_Mon_/*b*_Mon_ ratio (Figure 1c, top), and the inter-plane shear represented by the delta angle as described by Chung *et al*.^8^ (Figure 1c, bottom). As described in ref 11, these modes can be used to quantify the extent of monoclinic distortion as a function of temperature.^11^ The lattice parameter conversion between the two cells and the equations for the distortion modes can be found in S1; in a perfectly rhombohedral structure *a*_Mon_/*b*_Mon_ and delta will equal $\sqrt{3}$ and 90°, respectively.

Many of the underlying structural differences between LNO and NNO are thought to originate from off-stoichiometry and the antisite defects seen for LNO (collectively referred to as substitutional defects).^21–23^ Off-stoichiometry arises from the unavoidable loss of lithium due to its volatility during synthesis and this material is often referred to as Ni-excess with a Ni:Li ratio greater than one (Li_1–*x*_Ni_1+*x*_O_2_). Extra Ni occupy sites in the lithium layer (Ni_Li_^•^), which is accompanied by a reduced Ni^2+^ in the nickel layer (Ni_Ni_^′^) to balance charges (expressed using Kröger–Vink notation). An antisite defect corresponds to the stoichiometric switching of Li/Ni cations resulting in Ni^2+^ in the Li layer (Ni_Li_^•^) and Li^+^ in the nickel layer (Li_Ni_^″^), with an oxidized (formally) Ni^4+^ in the nickel layer (Ni_Ni_^•^) to balance charges. The formation of this defect has been ascribed to the similar ionic radii of Li^+^ (*r* = 0.76 Å) and Ni^2+^ (*r* = 0.69 Å). It is an entropically driven process that occurs during the high temperature synthesis of the material, arising from the incomplete conversion of a disordered cubic Li_*x*_Ni_1–*x*_O_1−δ_ intermediate to the ordered layered LiNiO_2_.^24,25^ Typically, LNO synthesized by solid-state methods (SS-LNO) contains a combination of both off-stoichiometry and antisite mixing, resulting in substitutional defects on the order of 1–5%. The wider impacts of the defects in LNO are the subject of considerable research, focusing, for example, on the short- and long-range structure,^26^ the effects on magnetic properties,^27^ and the influence of defects on diffusion and the electrochemical behavior of the material.^28,29^ The defects have been proposed to prevent any long-range JT ordering in work by Genreith-Schriever et al. where the defects were shown via *ab initio* molecular dynamics (AIMD) simulations to impede cooperative ordering by pinning the undistorted domains.^11^

Antisite defects are not observed in NNO due to the larger Na^+^ ionic radius. However, various studies have investigated the structure and properties of doped NaNiO_2_, whose results help to inform our understanding of the influence of substitutional defects and disorder in layered nickelates more broadly (including LiNiO_2_). For example, a study by Sada *et al*. showed that Mg-doping of NNO reduces the JT distortion and extent of monoclinicity, owing to the disruption of the long-range cooperative Jahn–Teller order.^30^ Additionally, earlier work by Delmas *et al*. found that the partial substitution of Co for Ni in NaNiO_2_ similarly results in the suppression of the cooperative JT distortion.^31^

Recently, Karger et al. employed low-temperature Na/Li ion-exchange to synthesize defect-free LNO (IE-LNO) by exploiting the defect-free nature of NNO.^32^ In this work, we investigate the short- and long-range structure of IE-LNO using solid-state nuclear magnetic resonance (ssNMR) spectroscopy, magnetic measurements, and X-ray and neutron diffraction, comparing our results with a typical SS-LNO sample. We observe that the high degree of ordering changes the electrochemical and magnetic properties of IE-LNO and demonstrate that the absence of substitutional defects leads to a monoclinic distortion at room temperature similar to that seen in NNO. A full understanding of the local and long-range structure of LNO is crucial to examining structure–property relations and can enable targeted research into methods to mitigate the degradation that originates from structural phase transitions on (de)­lithiation, ultimately, helping to produce commercially viable, high energy-density cathode materials.

## Experimental Methods

### LiNiO_2_ Synthesis

NaNiO_2_ was prepared by solid-state synthesis from a mixture of 1.1 equiv. NaOH (Sigma-Aldrich) and 1.0 equiv. Ni­(OH)_2_ (*d*_50_ = 4 μm, BASF SE) in oxygen (5 atm/h) at 500 °C for 12 h (with 3 K min^–1^ heating and cooling rates). Ion exchange of NaNiO_2_ to form IE-LiNiO_2_ was accomplished by heating a 1:1 (by weight) mixture of NaNiO_2_ and LiNO_3_ (Sigma-Aldrich) at 300 °C for 6 h. After cooling to room temperature, nitrate salt was removed by washing using an aqueous LiOH solution (0.025 M). Finally, the product was dried at 70 °C under dynamic vacuum, followed by sieving (45 μm) in an argon-filled glovebox. For more details, see refs 29 and 32. The lithium nitrate salt was unenriched so the IE-LNO sample will contain a natural abundance of ^6^Li and ^7^Li isotopes. The synthesis and characterization of the SS-LNO^33^ has been described in ref 33.

### Solid-State NMR

Samples were packed into 1.3 mm diameter magic angle spinning (MAS) rotors in an argon filled glovebox. ^7^Li NMR spectra were acquired with a 4.7 T Bruker Avance III spectrometer (200 MHz ^1^H Larmor frequency) with a spinning speed of 55 kHz. A magic-angle turning phase-adjusted sideband separation (MATPASS) pulse sequence was used with a π/4 pulse length of 0.5 μs. The peak of solid Li_2_CO_3_ at 0 ppm was used to reference ^7^Li chemical shift and the spectra were normalized based on number of scans and sample mass.

^23^Na NMR spectra were acquired with a Bruker Biospin Solid-State AV500 (500 MHz, 11.7 T) and a double resonance (HX) probe using a MAS speed of 50 kHz. The spectra were obtained with a π/4−π/2−π/4 rotor-synchronized spin echo pulse sequence with radiofrequency (RF) pulse lengths of 0.87 μs for π/4 and 1.74 μs for π/2 at a power of 50 W. A recycle delay of 50 ms was used to ensure that the ^23^Na signal had fully relaxed between pulses. The spectra were referenced to solid NaCl (7.2 ppm). Sodium quantification was performed by comparing the intensity of the spectrum to that of a known mass of solid NaCl and fitting was conducted using SOLA (more details in S3).

### Magnetic Measurements

Magnetic property measurements were performed using a Quantum Design Magnetic properties measurement system (MPMS3) using a superconducting quantum interference device (SQUID). Samples were wrapped in polyethylene film and loaded into MPMS plastic sample holders in an argon filled glovebox, and then mounted into the MPMS brass sample holder. DC magnetic susceptibility measurements were performed on the samples cooled at zero field (ZFC) at a range of temperatures (1.8–349.9 K) and under constant external field of 100, 1000, and 10,000 Oe. Fitting to the Curie–Weiss law was carried out using bespoke code written in Python.

### Electrochemical Characterization

The LNO powders were mixed with conductive carbon Super P (Cambridge Energy Solutions) and polyvinylidene fluoride (PVDF) binder (MTI, predissolved in 1-Methyl-2-pyrrolidinone, NMP, Sigma-Aldrich) in a weight ratio of 90:5:5 in a Thinky mixer at 2000 rpm. The slurry was cast on an aluminum foil with a doctor blade and transferred into a vacuum oven to be dried at 120 °C overnight. The 2032 coin cells (stainless steel coin cell parts from Cambridge Energy Solution) were then assembled in an argon-filled glovebox (O_2_ and H_2_O < 0.5 ppm), using 120 μL of LP57 electrolyte (1 M LiPF_6_ in ethylene carbonate (EC)/ethyl methyl carbonate (EMC) 3:7 vol %, Sigma-Aldrich), glass-fiber separators (MTI) and Li metal disc counter electrodes. Galvanostatic cycling was conducted at a C-rate of C/10 in a voltage window of 3.0–4.3 V. The currents were calculated based on the mass of active material, assuming a theoretical specific capacity of 274 mAhg^–1^.

### Neutron Powder Diffraction

Neutron diffraction measurements were performed on the GEM time-of-flight (TOF) powder neutron diffractometer at the ISIS pulsed spallation neutron source, Rutherford Appleton Laboratory, UK. Around 4 g of powder was packed into a ϕ = 8 mm vanadium can using indium and copper as sealants for low and high temperature, respectively. Data were recorded at 100, 300, and 500 K.

### Powder and Variable Temperature XRD

The powder sample was packed in a ϕ = 0.5 mm quartz glass capillary and sealed with two-component epoxy in an argon-filled glovebox. High-resolution powder synchrotron X-ray diffraction (SXRD) patterns were acquired on beamline I11 at the Diamond Light Source, UK.^34,35^ Multi-Analyzer Crystal (MAC) detectors were used for higher resolution measurements using a step-size of 0.001° (used for the 100 and 300 K combined X-ray and neutron refinements). Variable temperature measurements used a position-sensitive detector (PSD, Mythen2) for greater time resolution. The sample was first cooled from room temperature to 100 K, allowed to equilibrate, and then heated at a rate of 6 K/min from 100 to 500 K, with periodic measurements. For all, the beam energy of 15 keV (wavelength = 0.825 Å) was refined against a Si standard.

### TOPAS Academic for Structural Refinements

Rietveld refinements of the X-ray and neutron diffraction patterns were carried out using TOPAS (Academic) v7.^36,37^ Combined refinements of the two data sets were performed to resolve the atomic positions and site-occupancies with greater accuracy. An absorption correction was refined for the neutron data sets to account for the natural abundance ^6^Li in the sample. More details of the refinements are given in S9.

The VT XRD data was analyzed using a sequential Rietveld refinement in TOPAS. An initial structure was provided from the 100 K neutron/X-ray corefinement to act as the “seed”, and each pattern on heating was fitted in turn using the previous refined structural parameters as a starting point. The Na_Li_^*x*^ impurity was modeled with the same thermal parameters as the Li since they share a crystallographic site.

## Results

### Characterization of Defect-Free LNO

Ion-exchanged LNO (IE-LNO) was synthesized according to Karger et al.^32^ Preliminary Rietveld refinements using the conventional rhombohedral cell (*R*3̅*m*) and the synchrotron X-ray diffraction (SXRD) powder patterns were consistent with literature values (see S2). A *c*/*a* ratio of the lattice parameters of 4.941 was found, indicating a high degree of Li/Ni ordering.^2^ A combined refinement with X-ray and neutron powder diffraction (NPD) data was then performed since this provides extra sensitivity to the Li occupancy due to the large and negative neutron scattering length for Li (Li: *b* = −1.90 fm, *Z* = 3 vs Ni: *b* = 10.3 fm, *Z* = 28).^38^ The site occupancies were refined and yielded additional density in the lithium layer, which can be modeled by either 0.7 ± 0.1% Ni_Li_^•^ defects or 2.5 ± 0.3% Na_Li_^*x*^. ^23^Na ss-NMR confirmed the presence of Na in IE-LNO (see S3), a broad resonance, centered at approximately 1350 ppm, being observed. The large (hyperfine) shift of this resonance is close to that of NaNiO_2_ (1440 ppm),^30^ and consistent with the presence of residual sodium in the Li layer (Na_Li_^*x*^) due to incomplete ion-exchange during the synthesis. This value is within the sodium solubility limit of ≤5% and consistent with previous findings.^32,39,40^ There was also a 1.0 ± 0.2 wt % NiO impurity phase, likely present in the original material and likely also formed by surface reduction. In this section, the IE-LNO material will be compared to a SS-LNO with 3% substitutional defects (for more details on the SS-LNO sample see S4).

### ^7^Li NMR

The ^7^Li ssNMR of IE-LNO (Figure 2a) reveals a near symmetric major resonance at 710 ppm and a small diamagnetic peak at 0 ppm corresponding to surface lithium salts (such as Li_2_CO_3_, LiOH, LiHCO_3_, etc.). By contrast, SS-LNO shows a significantly broader and asymmetric resonance at 750 ppm along with additional peaks at 480 ppm and −90 ppm that have been previously assigned as arising from sites near antisite defects. Specifically, the resonance at −90 ppm arises from a Li_Ni_^″^ ion that is not near Ni_Li_^•^ while the broader 480 ppm resonance arises from a variety of environments including Li_Ni_^″^ ions with two or more next-nearest Ni_Li_^•^ ions, and Li_Li_^*x*^ ions that are adjacent to a Li_Ni_^″^ defect or Ni_Ni_^•^ with no unpaired electrons.^23^ Li in LNO twin boundaries has also been proposed to contribute intensity to this resonance.^41^ Crucially, the absence of the additional peaks in IE-LNO provides further evidence for the high degree of Li/Ni ordering and the lack of antisites.^23^ Given that the SS-LNO sample contains 2.6 ± 0.1% defects (from synchrotron X-ray diffraction (SXRD), see S4) and the ratio of the ^7^Li defect-related peak intensities (at 480 and −90 ppm) to the noise is approximately 30–50:1, an estimate for the upper bound of the defect concentration in IE-LNO of <0.1% can be obtained.

**Figure 2:**
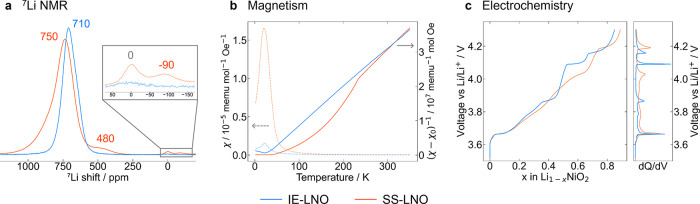
Defect-free nature of IE-LiNiO_2_. A comparison of IE-LNO (blue) and SS-LNO (orange). (a) ^7^Li MAS NMR, with an enlargement of the diamagnetic (∼0 ppm) and negative (−90 ppm) region. (b) SQUID magnetometry showing the ZFC magnetic susceptibility (dashed) and inverse susceptibility (solid) obtained with an applied field of 100 Oe. (c) Voltage vs Li content profiles obtained via electrochemical testing of LNO vs lithium metal cells with a C/10 charge and corresponding d*Q*/d*V* plot.

### Magnetism

Magnetic measurements also support the absence of substitutional defects, in particular the lack of nickel ions in the lithium layer. The magnetic susceptibility measurements of SS-LNO (solid orange line in Figure 2b) show a feature at approximately 240 K in the inverse susceptibility vs temperature plots, which has been associated with Ni_Li_^•^ defects and the accompanying Ni^3+^–O–Ni^2+^ superexchange interactions between Ni in the Ni layer (Ni_Ni_^*x*^) and Li layer (Ni_Li_^•^).^42–44^ This phenomenon is not observed in the ion-exchanged sample. Fits of the Curie–Weiss (CW) law to the experimental magnetization data at 10 kOe (more details in S5) yield a Weiss temperature of 26.7 ± 0.2 K for IE-LNO, consistent with <1% Ni_Li_^•^ defects using the trend established by Yamaura *et al*.^45^ By comparison, SS-LNO has a Weiss temperature of 74.6 K, corresponding to 3–4% defects. The magnetic moment per nickel was calculated to be 2.25 μ_B_ for IE-LNO, larger than the expected spin-only value of 1.73 μ_B_ for Ni^3+^, but within the range reported for previous studies of LNO. This elevated moment has been hypothesized to arise from unquenched orbital angular momentum contributions, mixing in of excited states, and/or spin disproportionation.^46,47^

### Electrochemistry

Electrochemical measurements also show notable differences between the two materials. The voltage profile of IE-LNO (Figure 2c) exhibits pronounced plateaus, also clearly seen as sharp peaks in the d*Q*/d*V* plot. The profile shares similarities with NNO, where a series of Na-vacancy and Ni charge ordered phases are formed, presumably made possible by the high degree of ordering and defect-free nature of NNO.^48–50^ This further supports the findings of XRD/NPD, ssNMR, and SQUID magnetometry, demonstrating the low concentration of substitutional defects in IE-LNO.

### Low-Temperature Structure: NPD and SXRD

Closer inspection of the room-temperature SXRD measurements shows broadening of select reflections, which is inconsistent with the previously reported rhombohedral structure of SS-LNO.^7^ To investigate this further we obtained time-of-flight (TOF) NPD and SXRD patterns over a wide range of temperatures: 100–500 K. The peak splitting was found to be most significant at low temperatures with clearly resolved splitting of the (101)_Rhom_ and (104)_Rhom_ Bragg peaks (*Q* = 2.56 and 3.08 Å^–1^ respectively) at 100 K, Figure 3. While the rhombohedral structure fails to capture the peak splitting, the splitting is consistent with IE-LNO adopting a monoclinic structure, with the (101)_Rhom_ becoming $\left(20\overset{-}{1}\right)$ _Mon_ and (110)_Mon_, and the (104)_Rhom_ becoming $\left(20\overset{-}{2}\right)$ _Mon_ and (111)_Mon_ reflections.

**Figure 3:**
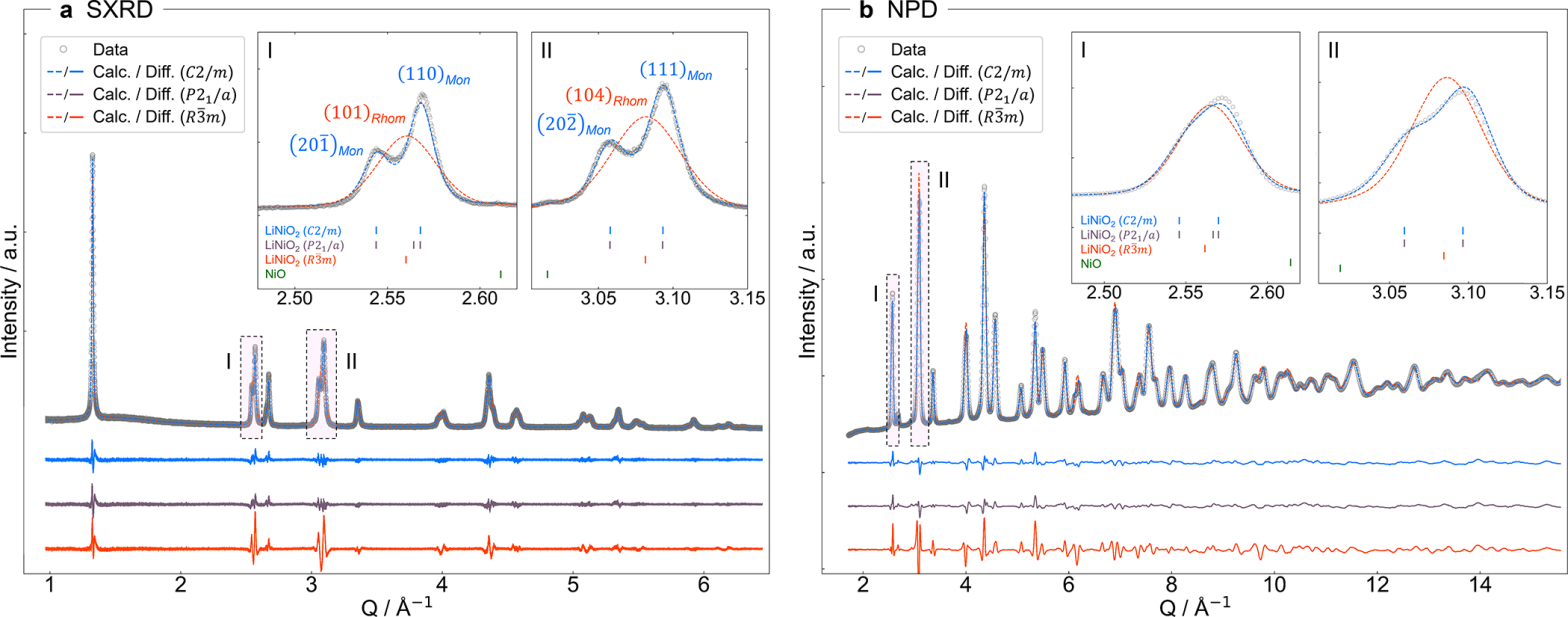
Combined X-ray and neutron diffraction refinements at 100 K. The fits resulting from combined Rietveld refinements using (a) SXRD and (b) TOF-NPD (shown for bank 4: 2θ = 50–74°, since this is the highest resolution bank with a *Q*-range appropriate to observe the two sets of reflections that correspond to monoclinic distortions). The data are shown by gray circles, the fits as dashed lines for *C*2/*m* (blue), *P*2_1_/*a* (purple), and *R*3̅*m* (orange) cells, and the differences are plotted below in the appropriate colors. The *C*2/*m* (blue) and *P*2_1_/*a* (purple) fits overlap. The insets (I and II) show the regions with the most significant peak splitting; the appropriate (hkl) indices for a monoclinic (blue) and rhombohedral (orange) cell are labeled in (a). The tick marks for each phase are shown below the trace in the insets.

No superstructure peaks were observed, ruling out the formation of an ordered charge-disproportionated structure. Ordering of the Ni^2+^ and Ni^4+^ in for example zigzag rows^51^ would result in a larger unit cell and thus extra peaks at lower *Q* in addition to the peak splitting. In the charge-ordered Na_2/3_NiO_2_, a series of superstructure peaks are observed from *Q* = 1.5–2.5 Å^–1^, but no such peaks are observed here for IE-LNO.^49^ A stripe ordering^51^ by contrast can be generated from a cell equal in size to the JT ordered unit cells resulting in no superstructure peaks, but the necessary oxygen and/or nickel displacements would generate additional peaks, which are not observed. We therefore consider the monoclinic distortion to be driven by ordering of the JT axes. To establish which ordering scheme is present and examine the 100 K structure of IE-LNO in more detail we carried out combined Rietveld refinements with the X-ray and neutron data using three different models: (i) the undistorted rhombohedral cell (*R*3̅*m*), (ii) a monoclinic cell (*C*2/*m*) exhibiting the collinear JT ordering, and (iii) a monoclinic cell (*P*2_1_/*a*) with a zigzag JT ordering. The results are shown in Figure 3 and summarized in Table 1.

**Table 1:** Structural Parameters from 100 K Neutron and X-ray Corefinements^a^

parameters		*C*2/*m*	*P*2_1_/*a*	*R*3̅*m*	*R*3̅*m* (monoclinic cell)
	*R*_wp_ [%]	2.828	2.827	5.775	
(unit cell)	*a* [Å]	5.00197(6)	5.00216(5)	2.87396(4)	4.97784(7)*
	*b* [Å]	2.86069(3)	2.86078(3)		2.87396(4)*
	*c* [Å]	5.02713(10)	5.02760(5)	14.1808(2)	5.00970(6)*
	β [°]	109.9180(11)	109.9163(11)		109.3545(4)*
	*formula volume* [Å^3^]	33.7945(5)	33.7976(5)	33.8150(11)	
	*a*_Mon_/*b*_Mon_	1.74852(2)	1.74853(2)	$\sqrt{3}=1.7321\cdot \cdot \cdot$	
	δ [°]	90.5481(12)	90.5476(11)	90.0	
(Ni–O bonds)	*s* [Å]	1.95733(19) x4	1.944(4) x2		
	*m* [Å]		1.970(4) x2	1.9694(3) x6	
	*l* [Å]	1.9880(4) x2	1.9883(4) x2		

a Results of Rietveld co-refinements with the *C*2/*m*, *P*2_1_/*a* and *R*3̅*m* cells, displaying the *R*_wp_, unit-cell parameters (including the degrees of monoclinic distortion: *a*_Mon_/*b*_Mon_ and the δ angle), and the Ni–O bonds lengths. For the *R*3̅*m* structure, the unit cell has also been converted to an undistorted monoclinic setting (denoted by a *) as per the relations outlined in S1. The atomic positions for each structure are tabulated in S6.

The *R*3̅*m* structure does not fit the data well at 100 K, with the higher symmetry structure being inconsistent with the observed peak splitting. By contrast, both monoclinic cells (*C*2/*m* and *P*2_1_/*a*) provide good fits to the data and capture the peak splitting (Figure 3). A closer inspection of the two resulting monoclinic structures reveals, however, that the two refinements had converged to the same structure within error. The *P*2_1_/*a* space group, which is appropriate for zigzag ordering, has a lower symmetry than the collinear *C*2/*m* structure but the additional degrees of freedom provided no noticeable improvement to the overall refinement, yielding an equivalent *R*_wp_ (2.827 and 2.828% respectively). This is further evidenced by the prevalence of systematic absences for the *P*2_1_/*a* cell (see S7), indicating that it is not necessary to reduce the symmetry further than *C*2/*m*, the highest-symmetry monoclinic space group. The refined atomic coordinates of the *C*2/*m* structure are consistent with a cooperative collinear JT ordering, with four short (1.96 Å) and two long (1.99 Å) Ni–O bond lengths per NiO_6_ octahedron.

Additional validation of the refined structure comes from examining the relationship between each JT ordering (collinear and zigzag) and the resultant in-plane *a*_Mon_/*b*_Mon_ ratio. In a collinear ordering (Figure 1b), the elongated O–Ni–O axes (bonds) of all NiO_6_ octahedra lie parallel and are aligned with the *a* direction of the monoclinic cell, while the short axes lie predominantly along *b*. This causes an increase in the *a*_Mon_/*b*_Mon_ ratio since the cell is stretched in *a* and compressed in *b* relative to the undistorted structure (*a*_Mon_/*b*_Mon_ > $\sqrt{3}$ ). By contrast, a zigzag ordering (Figure 1c) is comprised of alternating stripes of parallel elongated axes. The majority component of each elongated O–Ni–O axis lies along *b* of the monoclinic cell, with all octahedra possessing a short axis parallel to *a*. The zigzag ordering therefore results in a stretching in the *b* direction and a contraction in the *a* direction, overall reducing the *a*_Mon_/*b*_Mon_ ratio as compared to the undistorted cell (*a*_Mon_/*b*_Mon_ < $\sqrt{3}$ ). Refinements with both monoclinic symmetry cells (*C*2/*m* and *P*2_1_/*a*) led to a ratio greater than $\sqrt{3}$ , therefore consistent with collinear JT ordering.

### Phase Evolution from 100 to 500 K

Variable temperature (VT) XRD was then recorded during heating from 100 to 500 K to examine the nature of any structural phase transitions. Figure 4a shows a heatmap of the diffraction intensity, focusing on the *Q*-range containing the most significant peak splitting (full *Q*-range can be seen in S8) and highlighting the continuous merging of the peaks on heating. Figure 4b,c show the results of Rietveld refinements using the 500 and 100 K data sets, respectively. The low-temperature data shows a clear monoclinic distortion, however, at 500 K, both the monoclinic (*C*2/*m*) and rhombohedral (*R*3̅*m*) cells fit the pattern equivalently well, and so the material can be considered rhombohedral.

**Figure 4:**
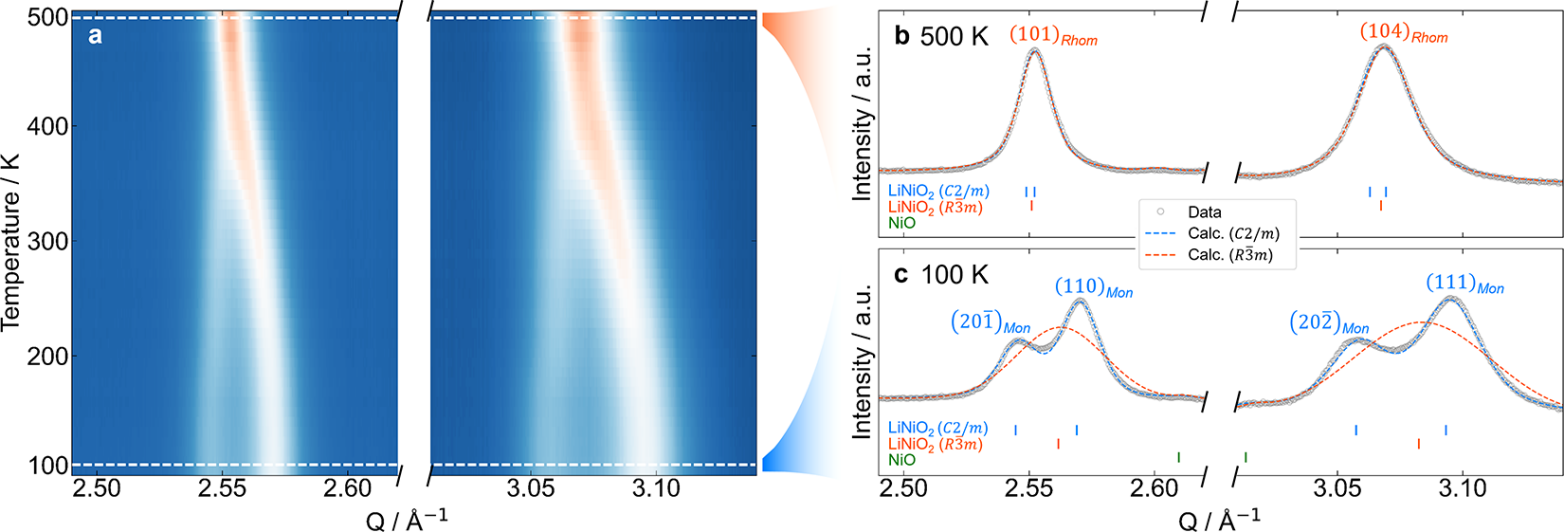
Monoclinic peak splitting in XRD patterns of IE-LNO as a function of temperature. (a) Heatmap of diffracted intensity for *Q*-range with most significant splitting. (b, c) Rietveld refinements of 500 and 100 K data sets, respectively, with monoclinic (*C*2/*m*, in blue) and rhombohedral (*R*3̅*m*, in orange) cells. The (hkl) indices of the splitting peaks are labeled for the monoclinic and rhombohedral cells.

Sequential refinements were performed on all intermediate data sets using the *C*2/*m* and *R*3̅*m* cells. The changes of the key parameters with temperature are shown in Figure 5 and are consistent with the results of NPD and SXRD corefinements (plotted as stars, more details in S9). Figure 5a shows the trend in the fit quality, *R*_wp_. The monoclinic cell remains stable at a value of ∼2.5% throughout, meaning that the cell provides an accurate fit to the structure across the whole temperature range. On the other hand, the rhombohedral cell fits the data comparatively poorly at low temperature, with an *R*_wp_ of 5.1%, reflecting the inability of the higher symmetry cell to generate the observed peak splitting. As the splitting decreases with increasing temperature, the *R*_wp_ of the fit using the rhombohedral cell continuously improves until ∼400 K when it plateaus to 2.7% and above 400 K the quality of the rhombohedral fit is comparable to the monoclinic cell. By considering that the monoclinic cell has significantly more degrees of freedom and yet offers little improvement to the fit quality, it is reasonable to conclude that the structure has become rhombohedral above 400 K. Characterization of a second synthetic batch of IE-LNO (summarized in S11–S13) yields similar results even though this sample contained more residual sodium (4.0 vs 2.5%).

**Figure 5:**
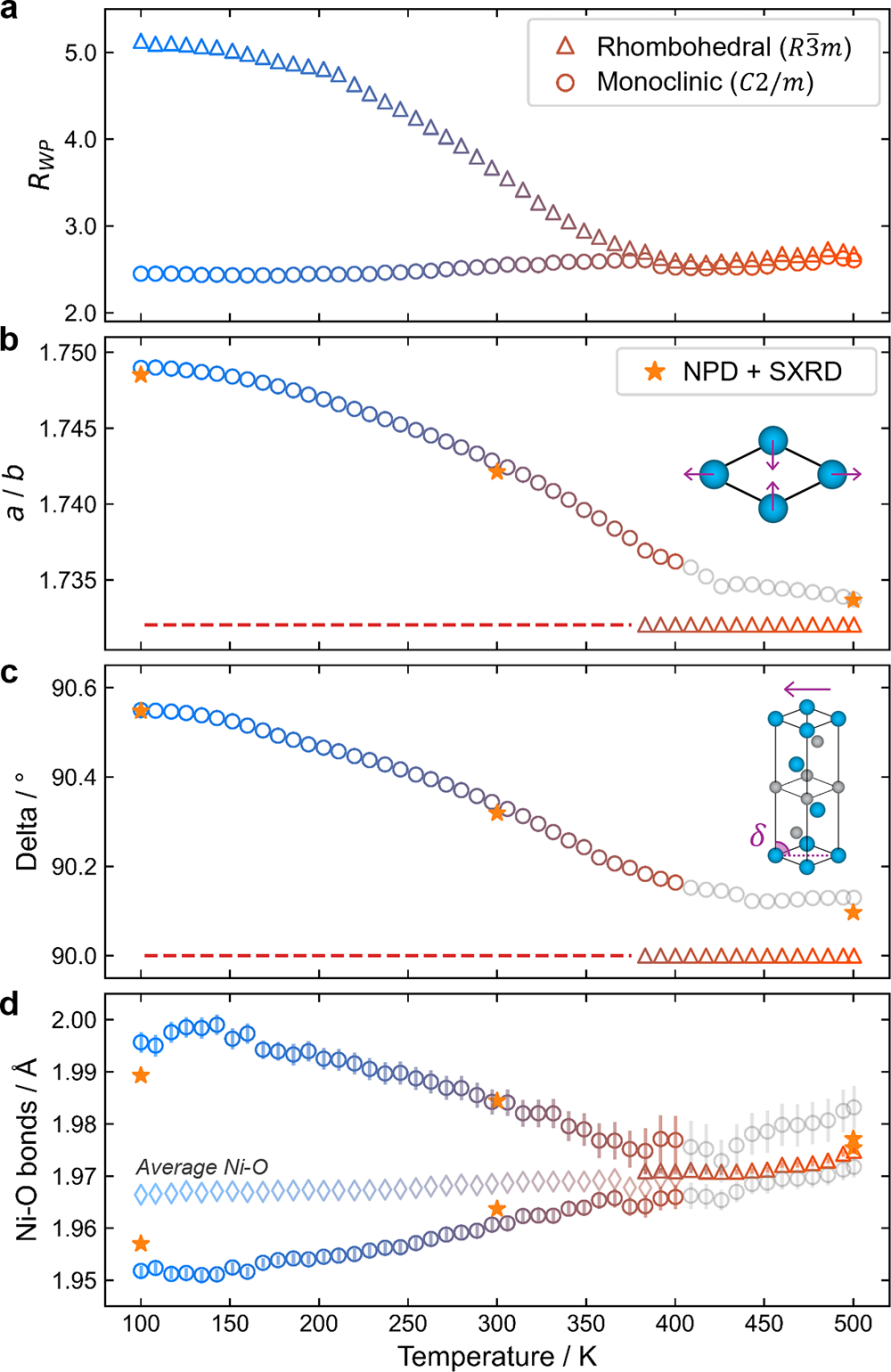
Outputs from VT XRD sequential refinements of IE-LNO. Trend in (a) *R*_wp_, (b) *a*_Mon_/*b*_Mon_ ratio, (c) delta angle, and (d) Ni–O bond lengths with rhombohedral (*R*3̅*m*, open triangles) and monoclinic (*C*2/*m*, open circles) symmetry cells. The average Ni–O bond lengths for the monoclinic phase are marked by diamonds. The results of NPD and SXRD combined refinements at 100, 300, and 500 K are shown with stars.

Figure 5b,c shows the trend of the monoclinic distortion modes both in-plane (*a*_Mon_/*b*_Mon_ ratio) and inter-plane (delta angle, illustrated inset in Figure 5c), and a steady decrease in the magnitude of distortion with temperature is observed. In a truly rhombohedral setting, the *a*_Mon_/*b*_Mon_ ratio would be $\sqrt{3}$ and the delta angle would be 90° (as indicated by the red dashed lines in each plot). The parameters do not fully converge on these undistorted values, but this is likely due to the inherent strain in the material, so that the monoclinic distortion captures some of the resulting peak broadening. Since at temperatures >400 K the *R*_wp_ of the two cells are equivalent and both accurately recreate the peak shapes and positions, this subtle monoclinic distortion can be considered as overfitting. This is consistent with the refinements of structures of the SS-LNO samples which, when refined with a monoclinic symmetry cell, also produce a small distortion.^11^ On the basis of the smooth and continuous decrease in the monoclinic distortion (measured by the *a*_Mon_/*b*_Mon_ ratio and delta angle as in Figure 5b,c, respectively) the phase transition from monoclinic (*C*2/*m*) to rhombohedral (*R*3̅*m*) on heating appears to be second-order. It is worth noting that since the *C*2/*m* space group of the low-temperature phase and the *R*3̅*m* of the high-temperature phase are related by a group-subgroup relationship, a second-order structural transition is allowed by symmetry.

### Jahn–Teller Distortions

The extent of the JT distortion as a function of temperature is displayed in Figure 5d. The low temperature structure exhibits a JT elongation with two Ni–O long bonds at ∼2.0 Å and four short bonds of ∼1.95 Å. With heating, these bond lengths smoothly converge to six essentially equivalent bond lengths above 400 K of ∼1.975 Å. A comparison of the structures from NPD-SXRD combined refinements at 100 and 500 K supports this trend in the JT distortion (Figure 6). Atomic displacement parameters (ADPs) are displayed as anisotropic ellipsoids and represent the thermal vibrations of the different atoms (for numerical values, see S10).

**Figure 6:**
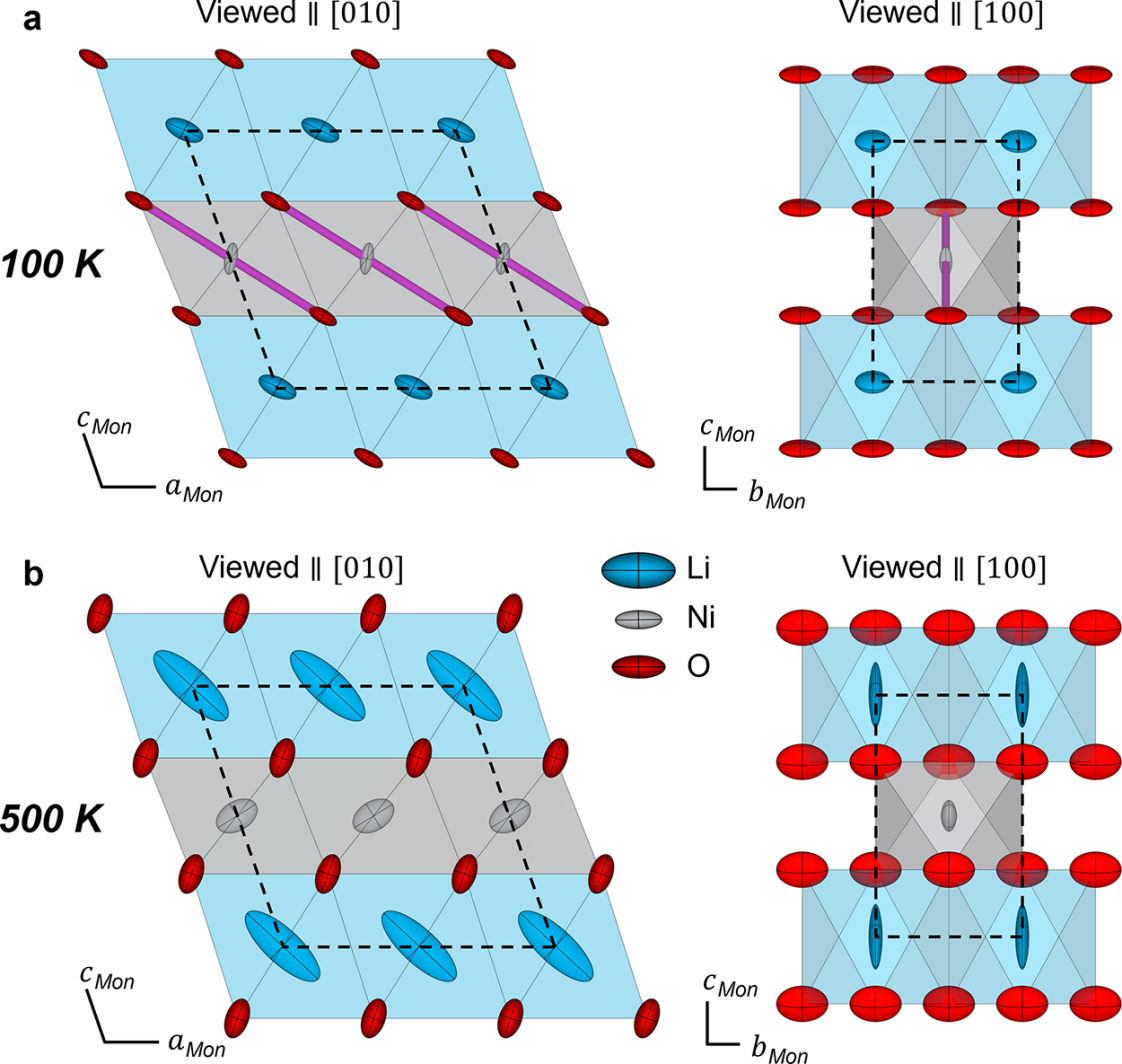
Structure of IE-LNO at 100 and 500 K. Crystal structure of IE-LNO determined by combined NPD and SXRD Rietveld refinement at (a) 100 K and (b) 500 K. The structures are shown viewed parallel to [010] and [100]. The ADPs for lithium (blue), nickel (gray), and oxygen (red) are displayed as anisotropic ellipsoids, the JT elongated Ni–O axes are highlighted in purple in (a), and the unit cell is shown by a black dashed line.

At 100 K (Figure 6a), the oxygens can be seen as thin displacement ellipsoids oriented along the direction of the JT elongated Ni–O bonds, suggesting significant vibrations along the JT axes. The lithium sites also exhibit flat ellipsoids, roughly coplanar with the oxygen, likely reflecting the correlated oxygen–lithium vibrations and phonon modes. By contrast, the nickel sites show rod-shaped displacements perpendicular to both oxygen and lithium and are comparatively more localized. At 500 K (Figure 6b), all displacements are enlarged, especially lithium, reflecting the increased thermal vibrations. The oxygen ADPs are no longer oriented parallel to the Ni–O bonds, consistent with the loss of JT distortions.

## Discussion

The long-range ordering presented in this work has not previously been observed in LNO and was enabled by the low defect-concentration of the material obtained from the ion-exchange synthesis method. In our prior work, AIMD simulations demonstrated that antisite defects pin the undistorted structure, so that there are only small, locally JT-distorted domains in regions or domains with no antisite defects.^11^ Subtle peak-broadening was seen in the low temperature XRD of a SS-LNO sample (containing ∼3% defects), consistent with only short-range monoclinic distortions.^11^ On heating, peak-sharpening was observed along with a gradual loss of distortions until ∼350 K, when refinements converged on a rhombohedral structure, consistent with a second-order phase transition. This trend parallels the findings of the current study, however, the low defect-concentration of IE-LNO facilitates long-range ordering and a more pronounced distortion. The observed peak splitting in IE-LNO allowed the nature of the monoclinic distortion to be investigated and revealed a cooperative collinear ordering of the JT distorted long axes, equivalent to the ordering seen in NNO. We propose that this collinear JT ordering is also present in solid-state synthesized LNO with the size of the domains governed by the defect concentration, with the defects disrupting the cooperative JT ordering over length-scales visible to bulk structure techniques. SS-LNO (with the *R*3̅*m* structure) has also been shown to undergo a transition to the *C*2/*m* monoclinic structure on lithium removal, for the nominal compositions Li_*y*_NiO_2_ (0.4 < *y* < 0.7) even when low concentrations of antisite defects are present in the pristine phase.^52–54^ The extent of monoclinicity found by Li *et al.* was similar to that of low-temperature IE-LNO (*a*_Mon_/*b*_Mon_ of 1.753 vs 1.749, respectively).^52^ Interestingly, in our recent ^7^Li NMR study of these monoclinic delithiated phases,^23^ we were no longer able to observe the resonance at −90 ppm assigned to Li ions in the nickel layer distant from Ni_Li_^•^ in this phase. Consistent with this prior and the current study, Hirano *et al*. observed that the structure of Li_0.5_Ni_1+*x*_O_2_ was monoclinic with *x* = 0.01, but that a rhombohedral structure was seen in X-ray and neutron diffraction with a greater density of substitutional defects (*x* = 0.05).^55^ A careful study of a series of Li_*y*–*x*_Ni_1+*x*_O_2_ samples with varying defect concentrations (*x*) would be required to examine the delithiated structures more comprehensively.

Although the monoclinic structure of IE-LNO is equivalent to the collinear JT ordered (*C*2/*m*) NNO phase, the magnitude of the monoclinic distortion at room temperature is considerably reduced. Both the *a*_Mon_/*b*_Mon_ ratio: 1.742 (LNO) vs 1.870 (NNO), and delta angle: 90.32° (LNO) vs 91.94° (NNO), are much closer to the undistorted values of 1.732 and 90° (NNO values calculated from parameters given by Dick et al.^18^). Furthermore, the phase transition in IE-LNO from the low temperature monoclinic (*C*2/*m*) structure to rhombohedral (*R*3̅*m*), appears to be second-order. The phase transition in NNO, by contrast, has been shown to occur at a higher temperature and to be first-order, exhibiting a clear mixed-phase regime between 460 and 505 K.^19^ One likely explanation for the different behavior is the presence of a small (*z* < 0.001), but nonzero degree of substitutional defects in the IE-LNO sample. Since the defects pin undistorted domains, even a concentration of only 0.1% could limit the cooperative domain size to ∼3 nm in diameter (approximated as 10x the Li–Li distance of 2.87 Å). The strain imposed by the domain boundaries and neighboring defects would limit the extent of lattice distortion and so the monoclinicity of the structure. The presence of nanoscale domains and defects can make a martensitic (diffusion-less) first-order phase transitions appear second-order as described by Reeve et al.,^56^ offering an explanation to the differing behavior in LNO and NNO. Additionally, the low levels of residual sodium present in the Li layers could apply a stress to the Ni slab due to the increased ionic radius of Na^+^ and thus also suppress JT distortions. However, very similar temperature-dependent structural behavior was seen between the two batches studied in this work despite different concentrations of residual Na, indicating that the influence of the residual sodium is likely smaller than that of Ni_Li_^•^ defects. A Ni_Li_^•^ substitutional defect is also associated with the formation of the non-JT-active Ni^4+^ ion, and thus there will also be additional electronic effects which suppress the JT distortions.

Furthermore, even assuming purely defect-free materials, differences in strain could occur due to the relative sizes of the LiO_6_ octahedra in LNO vs the NaO_6_ octahedra in NNO; the Li–O bonds in LNO are significantly shorter (2.12 and 2.11 Å) than the Na–O bonds in NNO (2.36 and 2.32 Å). With the smaller LiO_6_ it is more difficult to accommodate the frustration associated with the different orientations of the JT axes in LNO, resulting in a second order transition. The precise origin of this different behavior warrants further examination beyond the scope of this work, including an investigation of the domain sizes, elastic properties, and the impact of residual sodium in low-defect LNO.

## Conclusions

In conclusion, “defect-free” LNO has been recently synthesized by ion exchange from NNO and this work reveals and characterizes its monoclinic structure below 400 K. SQUID magnetometry and ^7^Li ssNMR spectroscopy exhibit no evidence of the features associated with Li/Ni substitutional defects and electrochemical cycling sees more pronounced voltage plateaus, characteristic of a highly ordered material. Neutron and X-ray diffraction reveal clear evidence of a monoclinic distortion and corefinements using the collinear and zigzag JT models converged on the same solution, namely a structure with a cooperative collinear JT distortion (with *C*2/*m* space group) that is equivalent to that of NNO. We propose that solid-state synthesized LNO samples also contain regions of collinear JT ordering at ambient and low temperatures with the domain-size and degree of distortion governed by the defect concentration. This work therefore provides an experimental answer to the structure of LNO, which has remained a puzzle within the literature for many decades.

## Supplementary Material



## Notes

Distortion mode equations, ^23^Na MAS NMR, Curie–Weiss fitting, tabulated refinement results, full stacks of SXRD/NPD patterns, and analysis of second IE-LNO synthetic batch (PDF)

## ○

Stanford Synchrotron Radiation Lightsource, SLAC National Accelerator Laboratory, Menlo Park, California 94025, United States
