# Supplementary material for: Collinear Jahn–Teller Ordering Induces Monoclinic Distortion in “Defect-Free” LiNiO2
Source: J Am Chem Soc. 2025 Jul 31;147(32):29042–51. doi: 10.1021/jacs.5c07435 (PMC12356588; doi:10.1021/jacs.5c07435)
Supplement: Supplementary file 1 [file ja5c07435_si_001.pdf]

Supplementary Information: Collinear Jahn-Teller Ordering Induces Monoclinic Distortion in “Defect-free” $LiNiO_2$

George S. Phillips[1,2], James M. A. Steele[1,3], Farheen N. Sayed[1,2], Leonhard Karger[4], Liam A. V. Nagle-Cocco[3,#], Annalena R. Genreith-Schriever[1,2], Gabriel E. Pérez[5], David A. Keen[5], Jürgen Janek[4,6], Torsten Brezesinski[4], Joshua D. Bocarsly[7], Siân E. Dutton[3], Clare P. Grey[1,2,*]

[1]*Yusuf Hamied Department of Chemistry, University of Cambridge, Lensfield Road, Cambridge, CB2 1EW, UK*

[2]*The Faraday Institution, Didcot, OX11 0RA, UK*

[3]*Cavendish Laboratory, University of Cambridge, JJ Thomson Avenue, Cambridge, CB3 0HE, UK*

[4]*Battery and Electrochemistry Laboratory (BELLA), Institute of Nanotechnology, Karlsruhe Institute of Technology (KIT), Kaiserstr. 12, 76131 Karlsruhe, Germany*

[5]*ISIS Neutron and Muon Source, Rutherford Appleton Laboratory, Harwell Science and Innovation Campus, Didcot, OX11 0QX, UK*

[6]*Institute of Physical Chemistry & Center for Materials Research (ZfM/LaMa), Justus-Liebig-University Giessen, Heinrich-Buff-Ring 17, 35392 Giessen, Germany*

[7]*Department of Chemistry and Texas Center for Superconductivity, University of Houston, Houston, Texas 77004, United States*

*Email: cpg27@cam.ac.uk

Contents

S1 – Monoclinic to rhombohedral lattice parameter conversion and distortion modes 3

S2 – Room-temperature XRD refinement, IE-LNO 4

S3 – ^{23}Na MAS NMR of IE-LNO and Na quantification 5

S4 – Room-temperature XRD refinement, SS-LNO 6

S5 – Magnetic characterisation and Curie Weiss fits 7

S6 – Atomic positions from 100 K refinements 8

S7 – Systematic absences for $\mathrm{P2_1/a}$ cell 9

S8 – Variable temperature XRD 10

S9 – SXRD/NPD co-refinements 11

S10 – Anisotropic ADPs 16

S11 – Structural refinement of IE-LNO ‘batch 2’ 17

S12 – Lattice parameter comparison of IE-LNO ‘batch 1’ and ‘batch 2’ 19

S13 – VT XRD comparison of IE-LNO ‘batch 1’ and ‘batch 2’ 20

S1 – Monoclinic to rhombohedral lattice parameter conversion and distortion modes

In order to compare the undistorted rhombohedral and distorted monoclinic structures it is necessary to understand the relationship between the two unit cells. The rhombohedral cell can be redrawn in a monoclinic setting (albeit with no monoclinic distortion) according to the following equations:

$$a_{Mon} = \sqrt{3}a_{Rhom} \quad [1]$$

$$b_{Mon} = a_{Rhom} \quad [2]$$

$$c_{Mon} = \frac{1}{3}\sqrt{3a_{Rhom}^2 + c_{Rhom}^2} \quad [3]$$

$$\beta = 90 + tan^{-1}\left(\frac{\sqrt{3}a_{Rhom}}{c_{Rhom}}\right) \quad [4]$$

It is possible to quantify the magnitude of the monoclinic distortion by considering the orthogonal distortion modes which break the rhombohedral symmetry. These modes consist of the in-plane distortion as given by the a_{Mon}/b_{Mon} ratio, and the inter-plane layer shearing quantified by the delta angle. The equations to calculate these modes are given below:

In-plane: $$\frac{a_{Mon}}{b_{Mon}} \quad [5]$$

Inter-plane: $$\delta \approx \beta - \sin^{-1}\left(\frac{a_{Mon}}{3c_{Mon}}\right) \quad [6]$$

S2 – Room-temperature XRD refinement, IE-LNO

Refinement of the IE-LNO sample using the typical rhombohedral ($R\bar{3}m$) cell. The residual in the two inset peaks will be discussed further.

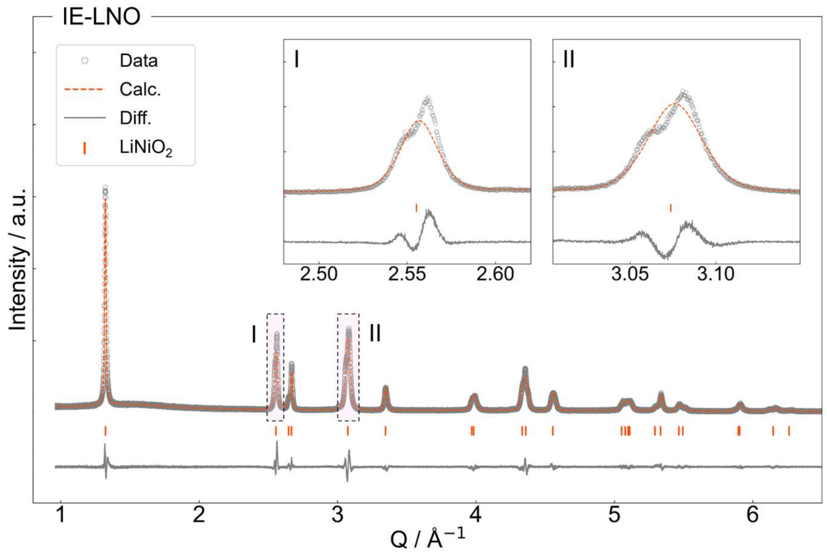

Figure S1 – Room-temperature XRD refinement. *Rietveld refinement using a rhombohedral $R\bar{3}m$ structure. The data is shown by grey circles, the fit as a dashed orange line, and the difference plotted below in grey. The Bragg reflections for the $LiNiO_2$ and NiO phases are labelled with blue and green ticks, respectively. The inset plots (labelled 'I' and 'II') show enlargements of the $(101)_{Rhom}$ and $(104)_{Rhom}$ Bragg peaks, respectively.*

Table ST1 – Room temperature XRD refinement, IE-LNO. *Overview of the parameters from Rietveld refinement with a rhombohedral $R\bar{3}m$ structure. Li_{Li} and Na_{Li} refer to Li and Na on the Li site, respectively.*

Parameter	Value	Parameter	Atom			
			Li_{Li}	Na_{Li}	Ni	O
R_{wp} [%]	10.696	Wyckoff site	3a	3a	3b	6c
a [Å]	2.878191(16)	*x*	0	0	0	0
c [Å]	14.22271(11)	*y*	0	0	0	0
c / *a*	4.9411(5)	*z*	0	0	0.5	0.24036(6)
V [Å^3]	102.036(1)	B_{eq} [Å^2]	0.39(4)	0.39(4)	0.324(6)	0.97(2)
		Occ.	0.975(3)	0.025(3)	1	1

S3 – ^{23}Na MAS NMR of IE-LNO and Na quantification

In order to identify the presence of sodium in the lithium layer (Na_{Li}^{x}), we used ^{23}Na ss-NMR (**Figure S2**). A very broad peak is observed at 1350 ppm, assigned to the Na_{Li}^{x} in the structure of IE-LNO (site 1 in **Figure S2b**). There is also a diamagnetic peak due to sodium impurity species, such as Na_2CO_3 (site 2 in **Figure S2b**).

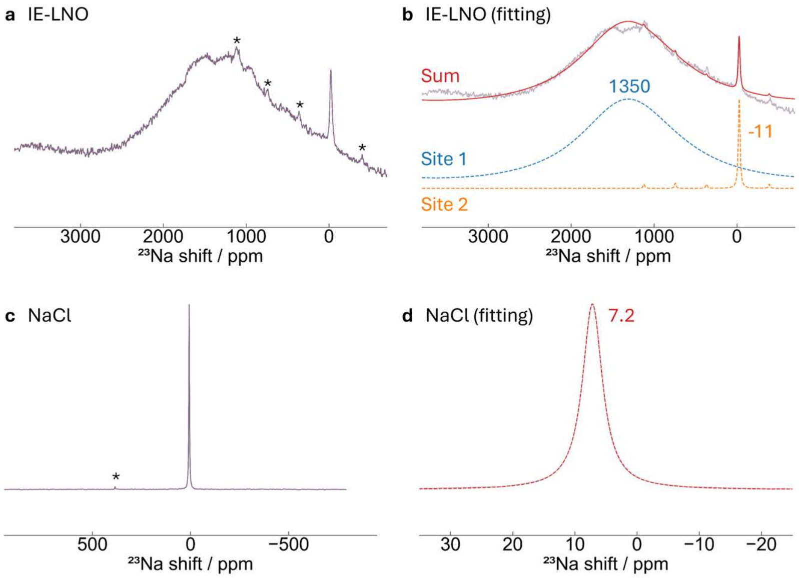

Figure S2 – ^{23}Na MAS NMR of IE-LNO and NMR fitting. *(a) ^{23}Na ss-NMR of IE-LNO measured with 50kHz MAS. The stars indicate the spinning sidebands of the diamagnetic peak at ~ 0 ppm. (b) The fit to the IE-LNO ^{23}Na spectrum (red line) with two Na environments, whose individual contributions are shown by the blue and orange dashed lines. (c) ^{23}Na ss-NMR of solid NaCl. The star indicates a spinning sideband. (d) The fit to the NaCl ^{23}Na spectrum (red dashed line) with one Na environment.*

Sodium quantification:

By fitting the ^{23}Na spectrum of IE-LNO (**Figure S2b**) and normalizing the integrated intensity using a NaCl reference sample (**Figure S2c** and **d**) it is possible to quantify the amount of Na in the IE-LNO material using the equation:

$$N_{sample} = \frac{I_{sample} \cdot MW_{sample} \cdot m_{NaCl} \cdot NS_{NaCl}}{I_{NaCl} \cdot MW_{NaCl} \cdot m_{sample} \cdot NS_{sample}}$$

Where N is the number of equivalent nuclei contributing to the signal, I is the integrated peak intensity, MW is the molecular weight, m is the sample mass and NS is the number of scans.

Using this equation, we calculate that the IE-LNO sample contains ~1.3% Na. There will be a moderate uncertainty in this value due to the poor signal-to-noise and difficulties with the baseline in the spectrum. Also, the very broad signal will correspond to a range of environments, so this calculation likely serves as an underestimation of Na content.

S4 – Room-temperature XRD refinement, SS-LNO

Refinement of the SS-LNO sample using the typical rhombohedral ($R\bar{3}m$) cell. Substitutional defects were refined to yield approximately 3% nickel-excess.

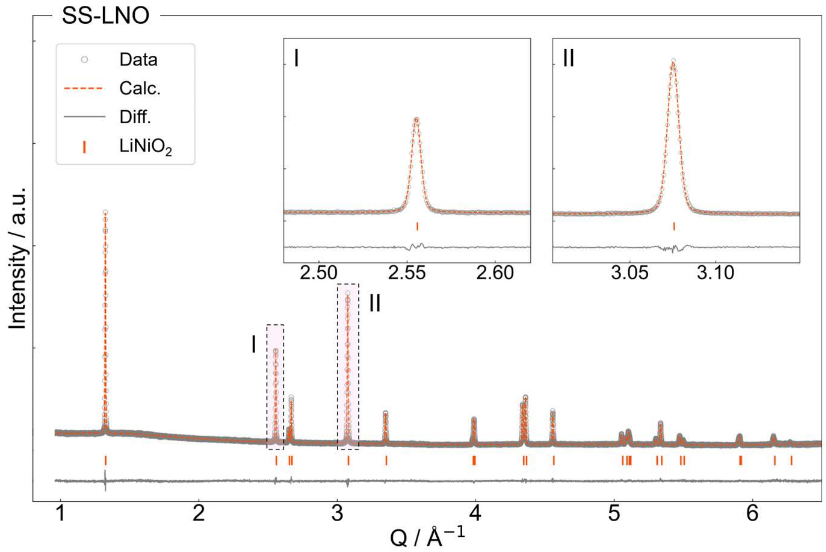

Figure S3 – Room-temperature XRD refinement. *Rietveld refinement using a rhombohedral $R\bar{3}m$ structure. The data is shown by grey circles, the fit as a dashed orange line, and the difference plotted below in grey. The Bragg reflections for the $LiNiO_2$ phase are labelled with orange ticks. The inset plots (labelled 'I' and 'II') show enlargements of the $(101)_{Rhom}$ and $(104)_{Rhom}$ Bragg peaks, respectively.*

Table ST2 – Room temperature XRD refinement, SS-LNO. *Overview of the parameters from Rietveld refinement with a rhombohedral $R\bar{3}m$ structure. Li_{Li} and Ni_{Li} refer to Li and Ni on the Li site, respectively.*

Parameter	***Value***	***Parameter***	***Atom***				
			Li_{Li}	Ni_{Li}	Ni_{Ni}	Li_{Ni}	***O***
R_{wp} **[%]**	5.128	***Wyckoff site***	3a	3a	3b	3b	6c
a **[Å]**	2.88210(2)	***x***	0	0	0	0	0
c **[Å]**	14.2207(14)	***y***	0	0	0	0	0
c / a	4.9362(5)	***z***	0	0	0.5	0.5	0.24150(11)
V **[Å³]**	102.299(2)	B_{eq} **[Å²]**	0.28(11)	0.28(11)	0.20(1)	0.20(1)	0.50(3)
		Occ.	0.974(1)	0.026(1)	1.000(5)	0.000(5)	1

S5 – Magnetic characterisation and Curie Weiss fits

The DC susceptibility data (**Figure S3**) shows paramagnetic behaviour above ~25 K, with a magnetic transition indicating the presence of magnetic ordering or glassiness at low temperature. The magnetic susceptibility was calculated in the low field limit ($\chi = dM/dH \approx M/H$). At temperatures well above the ordering temperature, the magnetic susceptibility (χ) can be fit to the Curie-Weiss law:

$$\chi(T) = \frac{C}{T-\theta} + \chi_0$$

Where C is the Curie constant, θ is the Weiss temperature, and χ_0 is a temperature independent parameter which captures the diamagnetic contributions of the sample. In order to avoid the 'anomaly' at 240 K arising from the presence of $Ni_{Li}^{\bullet}$ defects, the 10 kOe datasets were fitted from 250-350 K (**Figure S3**).

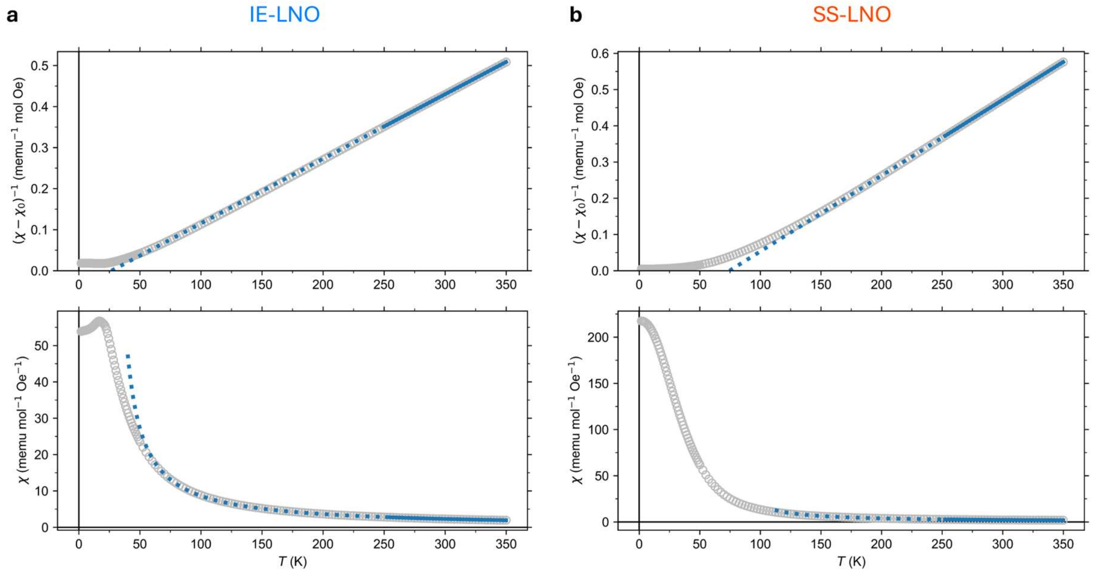

***Figure S4 – Curie-Weiss fits** for (a) IE-LNO and (b) SS-LNO from ZFC inverse magnetic susceptibility with an applied field of 10 kOe.*

S6 – Atomic positions from 100 K refinements

The atomic positions, thermal parameters, and occupancies for the $C2/m$ (**Table ST3**), $P2_1/a$ (**Table ST4**), and $R\bar{3}m$ (**Table ST5**) structures from SXRD and NPD combined refinements at 100 K. Li_{Li} and Na_{Li} refer to Li and Ni on the Li site, respectively.

Table ST3 – Atomic positions for $C2/m$ structure.

Parameter	*Atom*			
	Li_{Li}	Na_{Li}	*Ni*	*O*
**Wyckoff site**	2a	2a	2d	4i
x	0	0	0	0.23996(8)
y	0	0	0.5	0
z	0	0	0.5	0.72364(5)
B_{eq} [Å^2]	0.68(2)	0.68(2)	0.263(4)	0.621(3)
Occ.	0.975(3)	0.025(3)	1	1

Table ST4 – Atomic positions for $P2_1/a$ structure

Parameter	*Atom*			
	Li_{Li}	Na_{Li}	*Ni*	*O*
**Wyckoff site**	2a	2a	2d	4f
x	0	0	0.5	0.23993(8)
y	0	0	0	-0.006(2)
z	0	0	0.5	0.72363(5)
B_{eq} [Å^2]	0.69(3)	0.69(3)	0.265(3)	0.610(5)
Occ.	0.975(3)	0.025(3)	1	1

Table ST5 – Atomic positions for $R\bar{3}m$ structure

Parameter	*Atom*			
	Li_{Li}	Na_{Li}	*Ni*	*O*
**Wyckoff site**	3a	3a	3b	6c
x	0	0	0	0
y	0	0	0	0
z	0	0	0.5	0.24147(3)
B_{eq} [Å^2]	0.52(2)	0.52(2)	0.209(2)	0.729(9)
Occ.	0.975(3)	0.025(3)	1	1

S7 – Systematic absences in $P2_1/a$ cell

The $P2_1/a$ cell generates systematic absences, where allowed reflections see no intensity, displayed here for the 100 K refinement.

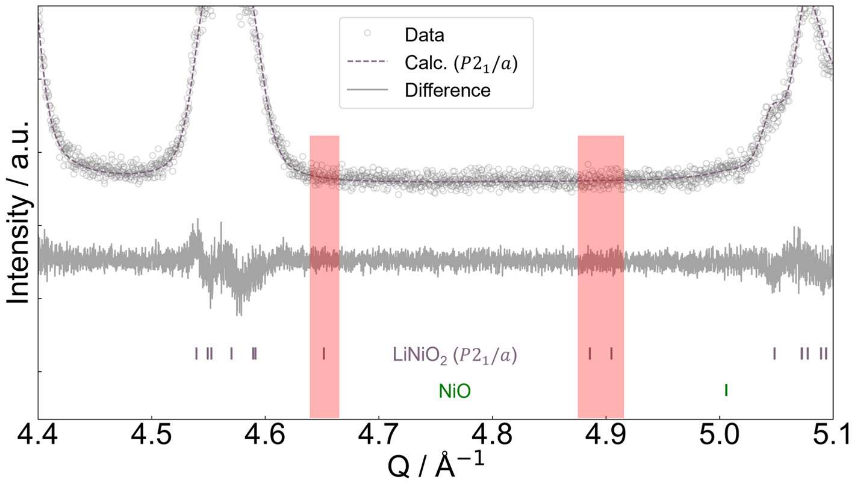

Figure S5 – Systematic absences in $P2_1/a$ cell. *Rietveld refinement of 100 K XRD pattern with a $P2_1/a$ cell, cropped to highlight systematic absences (red boxes). The data, refinement and difference are shown by grey circles, a purple dashed line and a grey line respectively. The tick marks for the $LiNiO_2$ phase (purple) and NiO impurity (green) are indicated below.*

S8 – Variable Temperature XRD

The variable temperature (VT) XRD patterns during heating from 100 – 500 K are presented below for a wider *Q*-range than in the main text (where the peak splitting is shown at $Q \sim 2.56$ and 3.08 $Å^{-1}$).

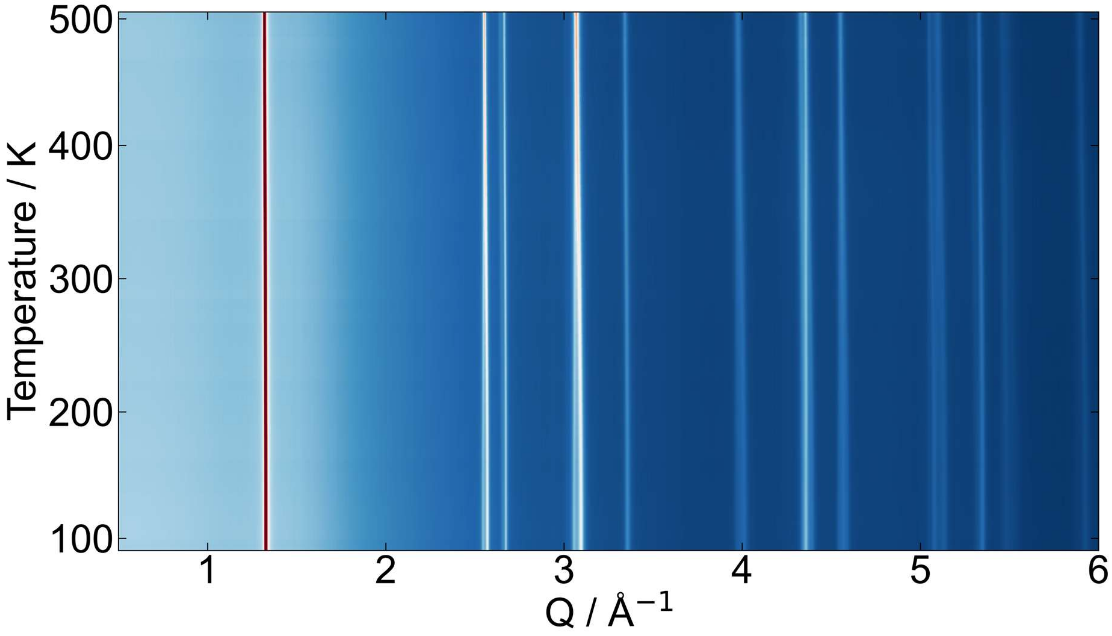

Figure S6 – VT XRD heatmap. *XRD intensity during heating from 100 – 500 K.*

S9 – SXRD/NPD combined refinements

Combined Rietveld refinements of the X-ray and neutron data (6 banks) were performed on the 100, 300, and 500 K datasets. The refinements were performed so that the neutron banks collectively were weighted equally to the X-ray data (a relative weighting of 0.1 for each neutron bank and 0.6 for the X-ray data), since this provided an equivalent goodness-of-fit (GOF) for the higher resolution neutron banks and the X-ray data. An absorption correction was refined to account for the natural abundance ^{6}Li in the sample. There are a very small set of peaks from the Vanadium can and furnace (labelled with a star in **Figure S7**). They are only just visible above the background and well separated from sample peaks so were therefore disregarded in the refinement.

Table ST6 – 100 K refinement results for $C2/m$ structure. *Combined SXRD and NPD Rietveld refinement results.*

Parameter	*Value*	*Parameter*	*Atom*			
			Li_{Li}	Na_{Li}	*Ni*	*O*
a **[Å]**	5.00197(6)	***Wyckoff site***	2a	2a	2d	4i
b **[Å]**	2.86069(3)	***x***	0	0	0	0.23996(8)
c **[Å]**	5.02713(10)	***y***	0	0	0.5	0
**β [°]**	109.9180(11)	***z***	0	0	0.5	0.72364(5)
V **[Å^3]**	67.521(2)	B_{eq} **[Å^2]**	0.68(3)	0.68(3)	0.263(4)	0.621(3)
		Occ.	0.975(3)	0.025(1)	1	1

Table ST7 – 300 K refinement results for $C2/m$ structure. *Combined SXRD and NPD Rietveld refinement results.*

Parameter	*Value*	*Parameter*	*Atom*			
			Li_{Li}	Na_{Li}	*Ni*	*O*
a **[Å]**	*4.99990(8)*	***Wyckoff site***	*2a*	*2a*	*2d*	*4i*
b **[Å]**	*2.87000(5)*	***x***	*0*	*0*	*0*	*0.23999(7)*
c **[Å]**	*5.03424(9)*	***y***	*0*	*0*	*0.5*	*0*
**β [°]**	*109.652(2)*	***z***	*0*	*0*	*0.5*	*0.72338(3)*
V **[Å^3]**	*67.797(2)*	B_{eq} **[Å^2]**	*0.73(3)*	*0.73(3)*	*0.302(3)*	*0.636(4)*
		Occ.	*0.975(3)*	*0.025(1)*	*1*	*1*

Table ST8 – 500 K refinement results for $C2/m$ structure. *Combined SXRD and NPD Rietveld refinement results.*

**Parameter**	**Value**	**Parameter**	**Atom**			
			Li_{Li}	Na_{Li}	*Ni*	*O*
a [Å]	4.99789(15)	*Wyckoff site*	2a	2a	2d	4i
b [Å]	2.88722(6)	*x*	0	0	0	0.2410(2)
c [Å]	5.04559(15)	*y*	0	0	0.5	0
β [°]	109.367(4)	*z*	0	0	0.5	0.72270(7)(5)
V [$Å^3$]	68.390(4)	B_{eq} [$Å^2$]	2.09(6)	2.09(6)	0.606(4)	1.01(5)
		Occ.	0.975(3)	0.025(1)	1	1

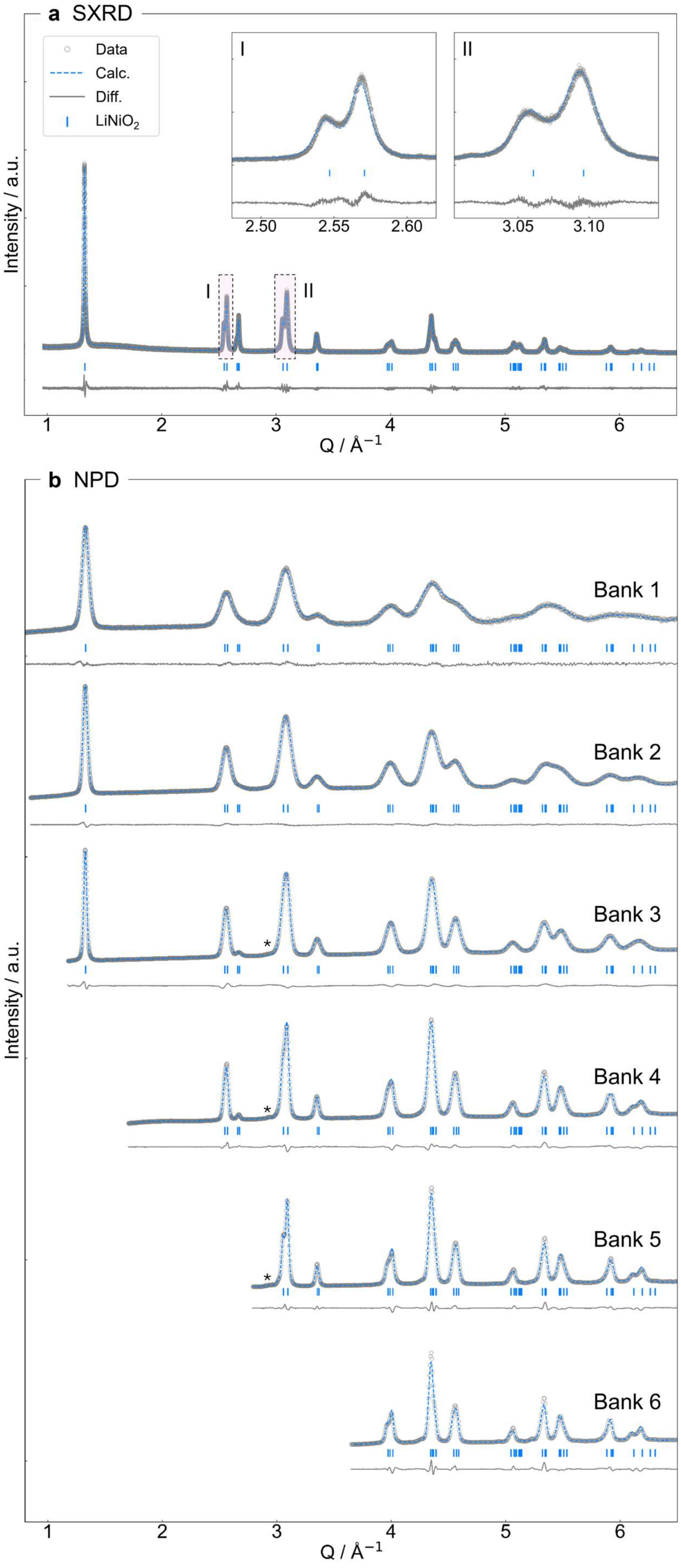

Figure S7 – SXRD/NPD combined refinement at 100 K. *(a) SXRD with λ = 0.825 Å and (b) TOF-NPD for detector banks 1-6. The data is shown by grey circles, the fit with $C2/m$ structure as a dashed blue line, and the difference plotted below in grey. The Bragg reflections for the $LiNiO_2$ phase are labelled with blue ticks. The insets in (a) show regions I and II with monoclinic peak splitting. The annotated stars on banks 3 – 5 indicate peaks from the Vanadium can and furnace.*

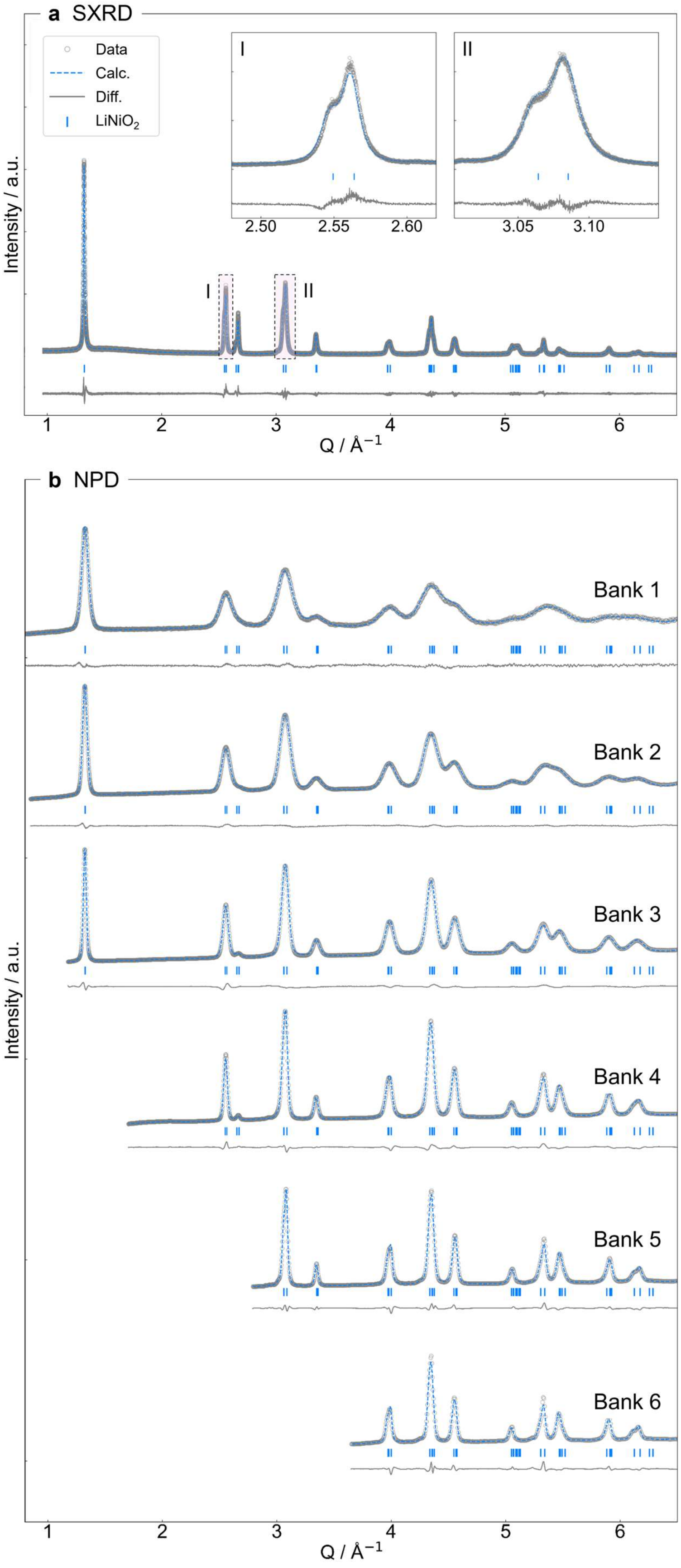

Figure S8 – SXRD/NPD combined refinement at 300 K. *(a) SXRD with λ = 0.825 Å and (b) TOF-NPD for detector banks 1-6. The data is shown by grey circles, the fit with* $C2/m$ *structure as a dashed blue line, and the difference plotted below in grey. The Bragg reflections for the* $LiNiO_2$ *phase are labelled with blue ticks. The insets in (a) show regions I and II with monoclinic peak splitting.*

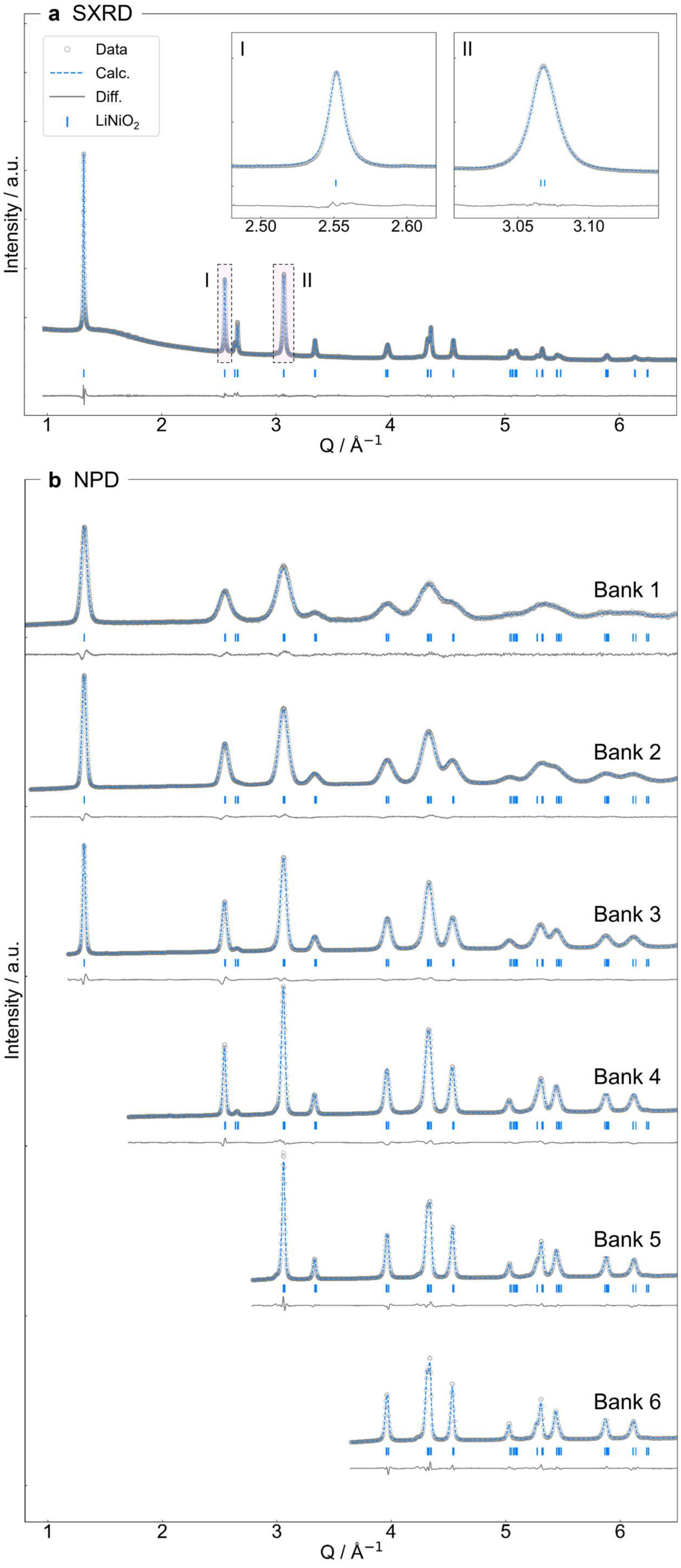

Figure S9 – SXRD/NPD combined refinement at 500 K. *(a) SXRD with λ = 0.825 Å and (b) TOF-NPD for detector banks 1-6. The data is shown by grey circles, the fit with $C2/m$ structure as a dashed blue line, and the difference plotted below in grey. The Bragg reflections for the $LiNiO_2$ phase are labelled with blue ticks. The insets in (a) show regions I and II with monoclinic peak splitting.*

S10 – Anisotropic ADPs

Results of combined SXRD and NPD refinements of the 100 and 500 K datasets with anisotropic ADPs.

Table ST9 – ADPs for 100 K refinement

Site	*Atom*	*ADPs* [Å^2]						
		U_{11}	U_{22}	U_{33}	U_{12}	U_{13}	U_{23}	B_{iso}
**2a**	**Li/Na**	0.0074(9)	0.0109(8)	0.0045(6)	0	-0.0018(6)	0	0.678
**2d**	**Ni**	0.00270(13)	0.00133(8)	0.00733(11)	0	0.00347(10)	0	0.263
**4i**	**O**	0.00396(18)	0.0147(2)	0.00287(13)	0	-0.00152(13)	0	0.621

Table ST10 – ADPs for 500 K refinement

Site	*Atom*	*ADPs* [Å^2]						
		U_{11}	U_{22}	U_{33}	U_{12}	U_{13}	U_{23}	B_{iso}
**2a**	**Li/Na**	0.022(2)	0.0024(15)	0.0380(13)	0	-0.0161(18)	0	2.091
**2d**	**Ni**	0.0143(3)	0.00209(17)	0.00858(18)	0	0.0064(3)	0	0.606
**4i**	**O**	0.0071(5)	0.0226(5)	0.0112(3)	0	0.0062(4)	0	1.014

S11 – Structural refinement of IE-LNO 'batch 2'

Combined SXRD and NPD Rietveld refinement results for a second synthetic batch of IE-LNO.

Table ST11 – Room temperature refinement results for IE-LNO 'batch 2' with $C2/m$ structure

Parameter	***Value***	***Parameter***	***Atom***			
			Li_{Li}	***Na_{Li}***	***Ni***	***O***
R_{wp} [%]	4.537	**Wyckoff site**	2a	2a	2d	4i
a [Å]	5.0011(2)	*x*	0	0	0	0.2404(4)
b [Å]	2.86672(13)	*y*	0	0	0.5	0
c [Å]	5.0298(2)	*z*	0	0	0.5	0.7216(2)
**β [°]**	109.7491(17)	B_{eq} [Å^2]	1.13(4)	1.13(4)	0.313(2)	0.770(5)
V [Å^3]	67.787(5)	***Occ.***	0.960(2)	0.040(2)	1	1

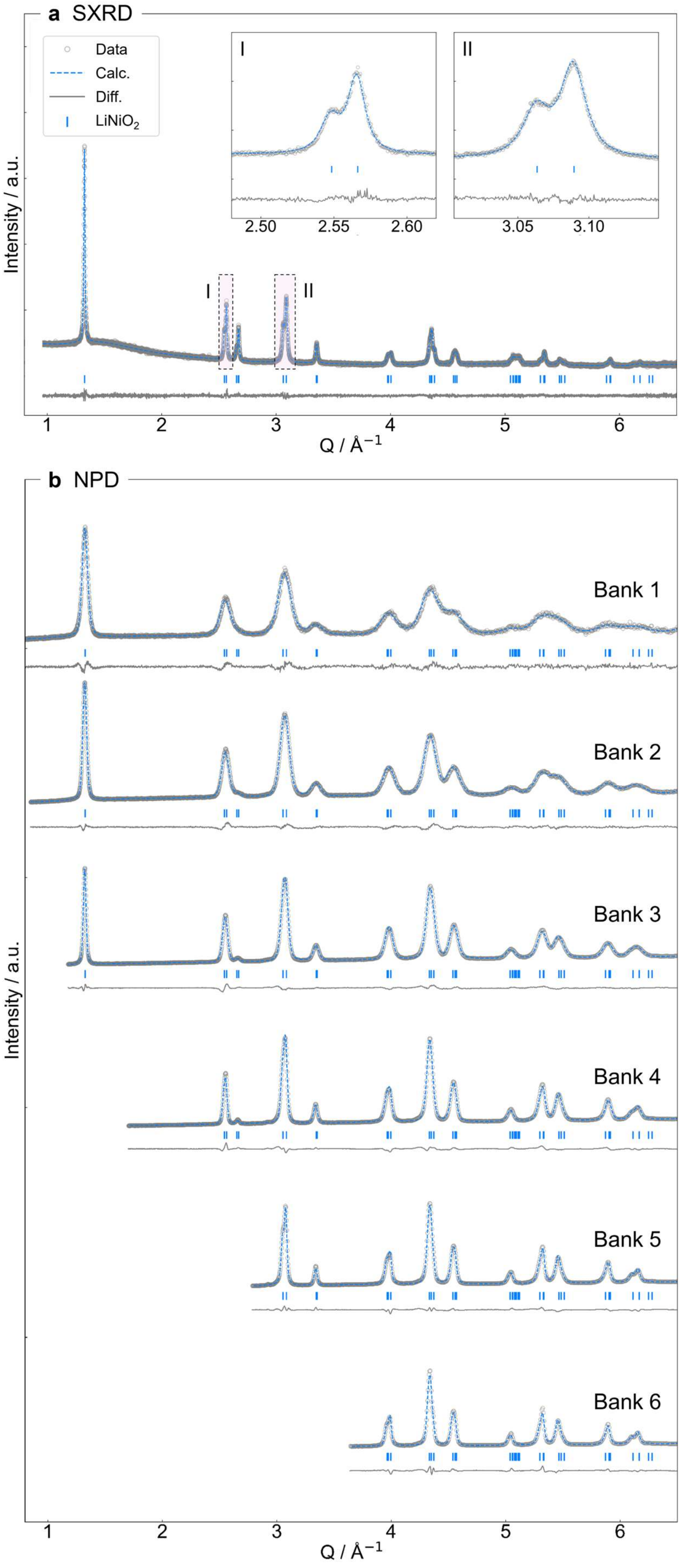

Figure S10 – SXRD/NPD combined refinement of IE-LNO 'batch 2' at room temperature. *(a) SXRD with $\lambda = 0.825$ Å and (b) TOF-NPD for detector banks 1-6. The data is shown by grey circles, the fit with $C2/m$ structure as a dashed blue line, and the difference plotted below in grey. The Bragg reflections for the $LiNiO_2$ phase are labelled with blue ticks. The insets in (a) show regions I and II with monoclinic peak splitting.*

S12 – Lattice parameter comparison of IE-LNO 'batch 1' and 'batch 2'

Table ST12 – Lattice parameters of IE-LNO 'batch 1' and 'batch 2'. *Results of the combined SXRD and NPD Rietveld refinement with $C2/m$ structure at room temperature.*

Parameter	*'Batch 1'*	*'Batch 2'*
***a* [Å]**	4.99990(8)	5.0011(2)
***b* [Å]**	2.87000(5)	2.86672(13)
***c* [Å]**	5.03424(9)	5.0298(2)
**β [°]**	109.652(2)	109.7491(17)
***V* [Å³]**	67.797(2)	67.787(5)
a_{Mon}/b_{Mon}	1.7418(4)	1.7446(1)
**δ [°]**	90.319(2)	90.393(2)
Na_{Li}	0.025(3)	0.040(2)

S13 – VT XRD comparison of IE-LNO 'batch 1' and 'batch 2'

Results of VT-XRD heating from 100 – 500 K for two different synthetic batches of IE-LNO looking at the peak splitting (**Figure S11**) and the variation of the monoclinic distortion modes: the in-plane a_{Mon}/b_{Mon} ratio (**Figure S12a**) and the inter-plane delta angle (**Figure S12b**).

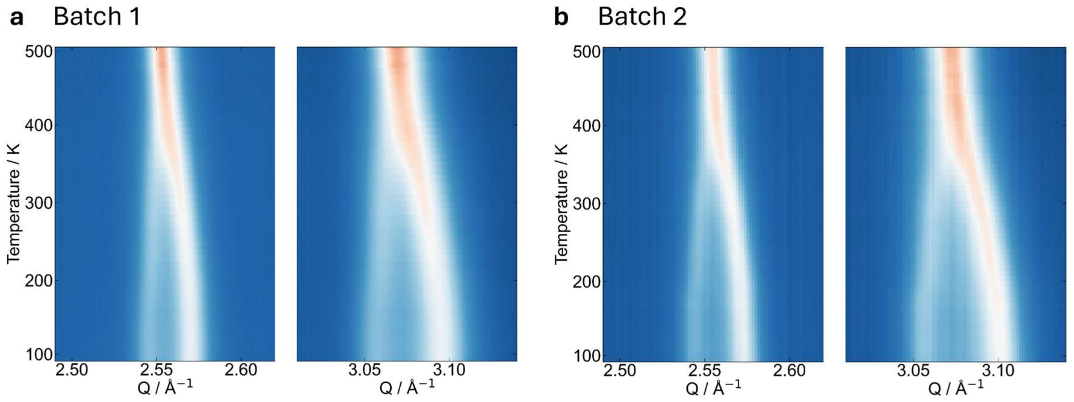

***Figure S11 – VT XRD heatmap comparison of IE-LNO batches.** (a) 'batch 1' and (b) 'batch 2' during heating from 100 – 500 K showing two Q-ranges with the most significant peak splitting (Q ~ 2.55 and 3.1 Å^{-1}).*

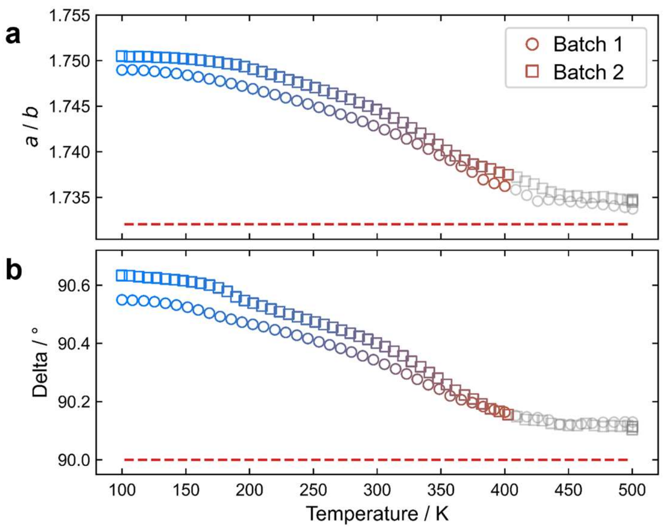

***Figure S12 – Monoclinic distortion modes from VT XRD sequential refinements comparing IE-LNO batches.** Variation of the monoclinic distortion modes: **(a)** in-plane a_{Mon}/b_{Mon} ratio and **(b)** inter-plane delta angle for IE-LNO 'batch 1' (circles) and 'batch 2' (squares).*
